# Targeting UDP‐glucose dehydrogenase inhibits ovarian cancer growth and metastasis

**DOI:** 10.1111/jcmm.15808

**Published:** 2020-09-07

**Authors:** Li‐Hsun Lin, Hsiu‐Chuan Chou, Shing‐Jyh Chang, En‐Chi Liao, Yi‐Ting Tsai, Yu‐Shan Wei, Hsin‐Yi Chen, Meng‐Wei Lin, Yi‐Shiuan Wang, Yu‐An Chien, Xin‐Ru Yu, Hong‐Lin Chan

**Affiliations:** ^1^ Institute of Bioinformatics and Structural Biology National Tsing Hua University Hsinchu Taiwan; ^2^ Institute of Analytical and Environmental Sciences National Tsing Hua University Hsinchu Taiwan; ^3^ Department of Obstetrics and Gynecology Hsinchu MacKay Memorial Hospital Hsinchu Taiwan; ^4^ Department of Medical Sciences National Tsing Hua University Hsinchu Taiwan

**Keywords:** EMT, ERK, metastasis, ovarian cancer, UGDH

## Abstract

More than 70% of patients with ovarian cancer are diagnosed in advanced stages. Therefore, it is urgent to identify a promising prognostic marker and understand the mechanism of ovarian cancer metastasis development. By using proteomics approaches, we found that UDP‐glucose dehydrogenase (UGDH) was up‐regulated in highly metastatic ovarian cancer TOV21G cells, characterized by high invasiveness (TOV21G^HI^), in comparison to its parental control. Previous reports demonstrated that UGDH is involved in cell migration, but its specific role in cancer metastasis remains unclear. By performing immunohistochemical staining with tissue microarray, we found overexpression of UGDH in ovarian cancer tissue, but not in normal adjacent tissue. Silencing using RNA interference (RNAi) was utilized to knockdown UGDH, which resulted in a significant decrease in metastatic ability in transwell migration, transwell invasion and wound healing assays. The knockdown of UGDH caused cell cycle arrest in the G_0_/G_1_ phase and induced a massive decrease of tumour formation rate in vivo. Our data showed that UGDH‐depletion led to the down‐regulation of epithelial‐mesenchymal transition (EMT)‐related markers as well as MMP2, and inactivation of the ERK/MAPK pathway. In conclusion, we found that the up‐regulation of UGDH is related to ovarian cancer metastasis and the deficiency of UGDH leads to the decrease of cell migration, cell invasion, wound healing and cell proliferation ability. Our findings reveal that UGDH can serve as a prognostic marker and that the inhibition of UGDH is a promising strategy for ovarian cancer treatment.

## 1 INTRODUCTION

Ovarian cancer is the leading cause of death in patients diagnosed with gynaecologic carcinoma. The American Cancer Society reported approximately 22 530 cases of ovarian cancer and 13 980 cancer‐related‐deaths in 2019, indicating a relatively high fatality rate. In the case report, over 60% of patients died from ovarian cancer, compared to 15% of those with breast cancer.^1^ This high mortality rate is due to the low diagnostic options in early stages. Over 70% of patients are at an advanced stage with regional or distant metastatic sites to the peritoneal cavity at the time of diagnosis.^2^ Thus, a detailed understanding of ovarian cancer metastasis is required to develop effective treatments.

Cancer metastasis is a complex process that involves multiple steps. It is caused by the ability of cancer cells to separate from the primary tumour, migrate to distant sites through the bloodstream or lymph system and form secondary tumours. Metastatic tumours are also responsible for higher rates of mortality in most cancers rather than primary tumours.^3^, ^4^ In the initial step of the metastasis cascade, cancer cells lose their cell‐cell connections and shed from primary tumour sites to invade the circulatory system. This step is triggered by a crucial process named as the epithelial‐mesenchymal transition (EMT). In EMT, cancer cells can leave their primary sites and invade the bloodstream. Thus, cancer cells lose their epithelial traits and enhance the mesenchymal phenotype to accomplish metastasis. During this period, metastatic cells gain the ability to degrade the extracellular matrix.^4^ These complex programs are co‐ordinated by numerous EMT‐inducing factors, such as SNAI1, SIP‐1 and Twist.^5^ Despite the great efforts, our understanding of metastasis progression is limited, and further studies of the detailed mechanisms of its regulation are required.

Glycosphingolipid (GSL) is a glycolipid on the cell membrane and plays numerous roles in cells, including cell adhesion, development, differentiation, tumour progression and signal transduction.^6^ Among all GSLs, Globo H (GH) has been reported to be associated with cancer progression. GH belongs to GSLs with the molecular structure of polysaccharide linked to the ceramide lipid ^7^ and the carbohydrate structure of GH is Fuca(1‐2)Galb(1‐3)GalNAcb(1‐3)Gala(1‐4)Galb(1‐4)Glcb(1).^8^ GH is overexpressed in numerous cancers, including breast, colon, gastric, endometrial, lung, ovarian, pancreatic, prostate cancer but shows low expression in normal cells.^9^ A cancer‐specific therapeutic vaccine composed of synthetic GH conjugated with keyhole limpet haemocyanin was developed for breast cancer. A previous study used a GH‐specific antibody MBr1 to for immunohistochemical staining in small cell lung cancer and found that GH‐positive tumours are associated with a shorter survival time than GH‐negative tumours.^10^ Several studies also demonstrated that the expression of GH is related to cancer aggressiveness in breast cancer and small cell lung cancer. Another recent publication revealed that GH cause immunosuppression by reducing Notch‐1 signalling in human peripheral blood mononuclear cells and trigger translin‐associated factor X‐dependent angiogenesis.^9^

UDP‐glucose dehydrogenase (UGDH) is an enzyme that catalyses NAD^+^‐dependent two‐step oxidation of UDP‐glucose to generate UDP‐glucuronate, but its roles and detailed regulatory mechanisms in cancer progression remain unclear. UGDH participates in tumour formation and cancer migration in breast cancer, colorectal carcinoma, glioblastoma and lung cancer.^11^, ^12^, ^13^, ^14^ Previous studies also demonstrated that UGDH is regulated by transforming growth factor‐β pathway, including p38, extracellular signal‐regulated kinase (ERK), mitogen‐activated protein kinase (MAPK). Moreover, UGDH has been reported to regulate cell glycosaminoglycan (GAG) level by catalysing the conversion of UDP‐glucose to UDP‐glucuronate. Recently, Wang et al^14^ found that inhibition of UGDH led to degradation of SNA1 mRNA and impaired lung cancer migration.

The current clinical condition of ovarian cancer presents high mortality rate. Although there are several improved treatments of ovarian cancer, including the platinum‐based chemotherapy and the cytoreductive surgery, the 5‐year overall survival of advanced ovarian cancer is approximately 30%.^15^ The clinical report implied that the ovarian cancer metastasis leads to the poor prognosis. Hence, the understanding of ovarian cancer metastasis mechanism is an urgent issue for improving the ovarian cancer treatment. To investigate the molecular mechanism of ovarian cancer metastasis, we analysed global protein changes between low invasiveness TOV21G cell line (TOV21G^LI^) and highly invasive cell line (TOV21G^HI^) by high‐through put proteomic approaches. In the obtained protein profile, we observed the overexpression of UGDH in TOV21G^HI^ cells. In the present study, we investigate the role and detailed mechanism of UGDH activity in ovarian cancer metastasis development. Our results provide insights into the identification of both new promising biomarkers and efficient therapeutic targets for ovarian cancer treatment.

## 2 MATERIALS AND METHODS

### 2.1 Chemicals and reagents

Lipofectamine^®^ RNAiMAX transfection reagent was purchased from Invitrogen (Thermo Fisher scientific Inc), and OPTI‐MEM was purchased from Gibco (Thermo Fisher scientific Inc). MTT (3‐(4,5‐Dimethylthiazol‐2‐yl)‐2,5‐diphenyltetrazolium bromide) was purchased from USB Corp. Propidium iodide (PI) was purchased from Sigma‐Aldrich. Anti‐Globo H primary antibody Mbr‐1 was obtained from Dr Chih‐Long Chang, Taipei MacKay Memorial Hospital, Taiwan. Anti‐rabbit and antimouse immunoglobulin (Ig)G horseradish peroxidase (HRP) conjugated secondary antibodies were purchased from Jackson ImmunoResearch Laboratories, Inc. Anti‐rabbit and antimouse immunoglobulin (Ig)G FITC‐conjugated secondary antibody were purchased from SeraCare KPL. All Biochemicals, chemicals and reagents used in this study were of analytic grade.

### 2.2 Cell lines and cell cultures

Human ovarian cancer cell line TOV21G was obtained from Dr Chih‐Long Chang, Taipei MacKay Memorial Hospital, Taiwan and cultured in RPMI‐1640 medium containing 10% foetal bovine serum (FBS) (Gibco, Thermo Fisher scientific Inc), streptomycin (100 ug/mL), penicillin (100 IU/mL). Human ovarian cancer cell lines A2780 and HeyA8 were obtained from Prof. Yung‐Jen Chuang, National Tsing Hua University and both cells were cultured in RPMI‐1640 with 10% calf bovine serum (CBS) (GE Healthcare Bio‐Sciences), streptomycin (100 μg/mL), penicillin (100 IU/mL). All cells were incubated at 37°C with 5% CO_2_.

### 2.3 Flow cytometry and cell sorting

1 × 10^6^ cells were suspended and fixed with 4% paraformaldehyde (PFA)/PBS for 20 minutes. Cells then blocked with 3% bovine serum albumin (BSA) in PBS for 1 hour. Anti‐Globo H primary antibody Mbr‐1 diluted in 1:400 was used to incubate with blocked cells for 2 hours at 4℃. Cells then were washed with PBS for three times for probing with FITC‐conjugated secondary antibody. After incubation with secondary antibody for 1 hour at 4℃, cells were subsequently applied to Accuri^TM^C6 flow cytometer for analysis. For cell sorting, 1 × 10^7^ cells were applied to previous described procedures without fixing. After secondary antibody incubation, cells were immediately applied to BD FACSAria™ III cell sorter for the analysis and sorting. Secondary antibody‐incubated‐only cell was negative control. Cells expressing relative high level of GH were sorted and defined as GH+ cells, whereas cells with relative low level of GH were defined as GH cells.

### 2.4 2D‐DIGE analysis and protein identification of MALDI‐TOF MS

Prior to perform 2D‐DIGE, the cell pellets were solubilized in 2D‐DIGE lysis buffer (4% w/v 3‐((3‐cholamidopropyl)dimethylammonio)‐1‐propanesulfonate (CHAPS), 7 mol/L urea, 2 mol/L thiourea, 10 mmol/L Tris‐HCl (pH 8.3) and 1 mmol/L EDTA). Insoluble materials were removed by centrifugation at 16 300 *g* for 30 minutes at 4°C, and protein concentrations were determined using Bradford Coomassie Protein Assay Reagent (Bio‐Rad). Protein samples were labelled with N‐hydroxy succinimidyl ester‐derivatives of the cyanine dyes of Cy2, Cy3 and Cy5. To accelerate image matching and cross‐gel statistical comparison, a pool of all samples was also prepared and labelled with Cy2 at a molar ratio of 2.5 pmol Cy2 per microgram of protein as an internal standard for all gels. All samples were run in triplicate against the standard pool. Subsequently, the fluorescence 2DE was scanned directly between the low‐fluorescent glass plates using an Ettan DIGE Imager, and gel analysis was performed using DeCyder 2‐D Differential Analysis Software v7.0 (GE Healthcare) to detect, normalize and quantify the protein features in the images. Spots displaying a ≥ 1.5 average fold increase or decrease in abundance with a *P*‐value < .05 were selected for protein identification. Colloidal Coomassie blueG‐250 staining was used to visualize CyDye‐labelled protein features in 2DE followed by performing interesting post‐stained gel pieces for MALDI‐TOF MS identification. The detailed procedures for protein staining, in‐gel digestion, and MALDI‐TOF MS analysis, and the algorithm used for data processing have been described in previous publication.^16^, ^17^ Peaks in the mass range of m/z 800‐3000 were used to generate a peptide mass fingerprint that was searched against the Swiss‐Prot/TrEMBL database (released on February 2014) with 542503 sequences using Mascot software v2.5.0.1 (Matrix Science). The following parameters were used for the search: *Homo sapiens*; tryptic digest with a maximum of one missed cleavage; carbamidomethylation of cysteine, partial protein N‐terminal acetylation, partial methionine oxidation, partial modification of glutamine to pyroglutamate and a mass tolerance of 50 ppm. Identification was accepted based on significant MASCOT scores (*P* < .05), spectrum annotation and observed versus expected molecular weight and pI on 2DE as well as at least five peptides in each identified protein.

### 2.5 Cancer tissue microarray analysis and immunohistochemistry (IHC)

UDP‐glucose dehydrogenase expression level of tumour specimens was measured by using tissue microarray, BC11115b, purchased from US Biomax, Inc. To perform immunochemical staining, anti‐UGDH antibody (Abcam) was used for incubation. The staining in intensity of UGDH in tissue microarray was measured and was analysed by ImageJ software (National Institute of Health).

### 2.6 SiRNA design and transfection

For transient knockdown experiment, small interfering RNA (siRNA) against UGDH was purchased from Invitrogen (Thermo Fisher scientific Inc). The targeting sequence against UGDH was 5′‐GGGCCUAGUSUCUAUCAGACAAAUU −3′. When reaching 50% confluence, cells were transfected by using Lipofectamine^®^ RNAiMAX transfection reagent according to the manufacturer's instructions. In brief, cells were transfected with 40 nmol/L of UGDH siRNA in OPTI‐MEM medium containing RNAiMAX for 6 hours. Transfected cells then recovered in serum containing medium for at least 16 hours. The knockdown efficiency of siRNA was verified by immunoblotting.

### 2.7 Immunoblotting

Protein samples were separated by 12% SDS‐PAGE (sodium dodecyl sulphate polyacrylamide gel electrophoresis) and electroblotted onto polyvinylidene difluoride (PVDF) membranes (Pall Corp.). Blotted membranes were blocked with 5% (w/v) skimmed milk or 5% bovine serum albumin (BSA) in Tris‐based saline with 0.5% Tween‐20 (TBST) for 1 hour and then incubated with primary antibodies overnight at 4°C. After membranes were washed in TBST (4 × 10 minutes), the appropriate horseradish peroxidase‐coupled secondary antibodies were probed onto membranes for 1 hour at room temperature (Jackson ImmunoResearch Laboratories, Inc). The membranes then were washed in TBST (6 × 10 minutes), and the immunoprobed proteins were visualized by using enhanced chemiluminescence method and the signal was detected by ImageQuant LAS‐4000 (GE HealthCare Life Sciences).

### 2.8 Lentiviral constructs for expression of anti‐UGDH shRNA and establishing of stable knockdown cell line

The anti‐UGDH shRNA constructs, control plasmid (pLKO1) and lentivirus package plasmid (pMD.G, pCMVDR8.91) were purchased from RNAiCore, Academia Sinica, Taiwan. For producing shUGDH lentivirus, HEK‐293T cells were transfected withpLKO1, shUGDH constructs, pMD.G and PCMVDR8.91 plasmids by using X‐tremeGENE transfection reagent (Roche Diagnostics) when cells reach 70% confluency. The X‐tremeGENE transfection reagent and plasmids were diluted with OPTI‐MEM medium (Invitrogen, Thermo Fisher scientific Inc) After 24h transfection, the medium was refreshed and the supernatant, which contained lentivirus particles was collected after 72 hours and filtered through 0.45 μm filters. TOV21G cells were transduced with pLKO and shUGDH lentivirus particles at two MOI (multiplicity of infection) treatment levels in 2 mL of complete medium containing polybrene (8 μg/mL) and incubated at 37°C, 5% CO_2_ for 48 hours. The transduced TOV21G cells underwent antibiotics selection in RPMI‐1640 medium containing 1 μg/mL puromycin for 3 weeks before applied to functional assays.

### 2.9 Transwell migration and Matrigel^TM^ invasion assay

Transwell permeable inserts with 8 μm pore size PET membrane (COSTAR, Corning Inc) were used to measure cell migration and invasion ability. Briefly, 1 × 10^5^ cells with serum‐free medium were seeded in the upper chamber and the complete medium with 10% FBS were loaded in the lower chamber for attracting cell migration. Cells then incubated at 37℃ for 8 hours, and the migrated cells were stained by crystal violet for quantification. Cells were visualized under the optical microscope and were displayed at a magnification of 100×. For preparation of matrigel invasion assay, Matrigel^TM^ (BD Biosciences) was added to ice‐cold FBS‐free medium in the ratio of 1:3 and 50 μL of diluted Matrigel^TM^ was coated on the upside of insert. Next, 1.5 × 10^5^ cells were added to the insert coated with Matrigel^TM^ and incubated at 37℃ for 16 hours. Invaded cells were stained by crystal violet for quantification.

### 2.10 Scratch wound healing assay

Cell was seeded in two well culture insert (ibidi, Germany) at a density of 3 × 10^4^ cells per well in 12‐well plate. After 24 hours incubation at 37℃, the insert was removed and the well was filled with culture medium for monitoring the healing area. Time‐lapse measurements were taken by optical microscope (Carl Zeiss) at 0, 4, 8 and 12 hours. The quantification and analysis of healing area were performed by AxioVision Rel. 4.8 software (Carl Zeiss).

### 2.11 Cell proliferation (doubling time) assay

TOV21G, A2780 and HeyA8 cells were trypsinized and seeded into 96‐well plates at a density of 3000 cells/ well. After 24 hours of incubation at 37℃, 5% CO_2_ (for Day 0 experiment), medium was removed and cells were incubated with 100 μL of MTT solution (1 mg/mL) per well for 4 hours at 37℃. The supernatant then was removed, and 100 μL dimethyl sulfoxide (DMSO) was added per well to dissolve the insoluble formazan. The 96‐well plate was then shook for 3 minutes before the absorbance was measured by spectrophotometer at 570 nm. The cell growth rates were monitored by the same method in the following time‐points, Day1 (24 hours), Day2 (48 hours) and Day3 (72 hours), respectively. The proliferation rates were presented as value relative to Day 0.

### 2.12 Apoptosis detection assay and cell cycle assay by flow cytometry

Apoptotic cells were stained with Annexin V FITC detection kit (BD Bioscience) according to the manufacturer's protocol. Briefly, cells were trypsinized and washed with ice‐cold phosphate‐buffered saline (PBS) before suspended in Annexin V binding buffer. Staining of 10^5^ cells was performed with 5 μL Annexin V solution and 5 μL propidium iodide (PI) solution at room temperature for 15 minutes, and samples then were subsequently applied to flow analysis by Accuri C6 Flow Cytometry (BD bioscience) for collection the emission data from FL1 channel and FL2 channel. For cell cycle analysis, 2 × 10^6^ cells were collected and fixed in 70% ethanol at 4°C overnight. Cells were washed twice with PBS and stained with 500 μL of PI staining solution (50 μg/mL PI and 100 μg/mL RNase in PBS) for 30 minutes in dark. Stained cells were then performed cell cycle analysis using Accuri C6 Flow Cytometry by collecting emission data from FL2 channel at 575 nm.

### 2.13 Metastatic assay in xenograft transplantation of nude mice model

For determining tumour formation, xenograft transplantation was used with BALB/cAnN.Cg‐Foxnlnu/CrlNarl nude mice. All animal experiments were performed in accordance with the Institutional Animal Care and Use Committee (IACUC) guidelines and approved by the IACUC (Approval No.: 10 539) of National Tsing Hua University. For analyzing the effect of UGDH on tumor formation rate, UGDH stable knockdown cell line and control cell line were used. Cells in PBS were mixed 1:1 with Matrigel^TM^ and adjusted to 0.3 mL for the subcutaneous injection. Mice were monitored the formation of tumour every 4‐5days and killed at 5‐6 weeks post‐injection for observing final tumour growth.

### 2.14 Statistical analysis

Data are presented as the mean ± SEM. Differences between the experimental groups were assessed using a paired Student's *t* test or a one‐way ANOVA followed by Tukey's multiple comparison test. Test results with *P* < .05 were considered statistically significant.

## 3 RESULTS

### 3.1 Identification of UGDH in highly invasive ovarian cancer cell line via proteomic analysis

**Figure 1:**
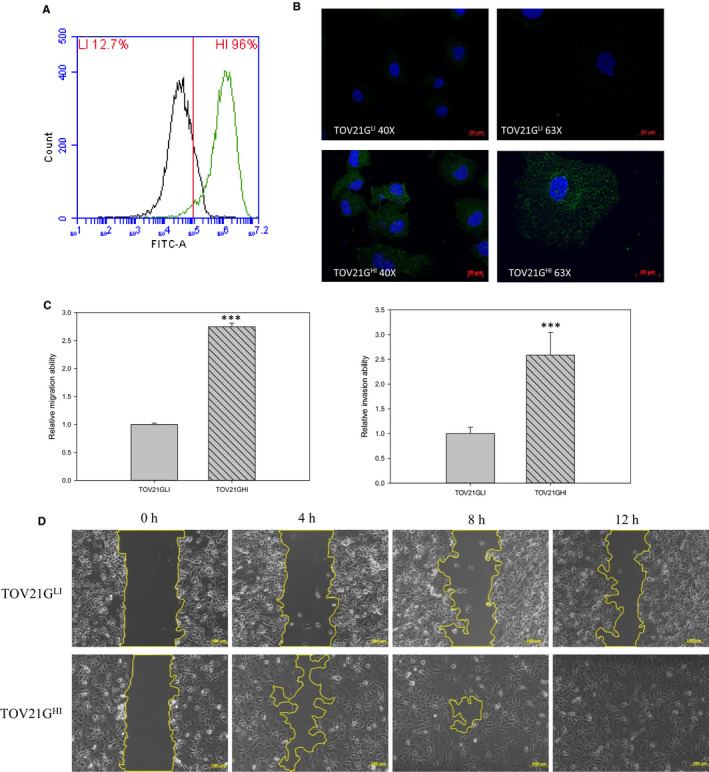
Isolation of highly invasive ovarian cancer cells according to the expression level of Globo H. GH‐specific antibody Mbr‐1 was used to detect GH expression in TOV21G^LI^/TOV21G^HI^ cells via flow cytometry and immunofluorescence (IF). A, Cells were treated with anti‐Globo H antibody followed by the FITC‐conjugated secondary antibody. Stained cells were analysed by flow cytometry by detecting FITC signal. B, TOV21G^LI^ and TOV21G^HI^ cells were incubated with anti‐Globo H antibody followed by FITC‐conjugated secondary antibody. DAPI was used for nuclear staining. The representative images are displayed at 40× and 63× magnification using fluorescence microscopy. C, Right panel: transwell invasion assay with matrigel pre‐coated condition was utilized to measure the invasive ability of TOV21G^LI^ and TOV21G^HI^ cells. Left panel: transwell migration assay was used for monitoring migration ability of TOV21G^LI^ and TOV21G^HI^ cells. The migration and invasion abilities were quantified by dissolving the cells stained with crystal violet on the underside of the membrane. Absorbance values were normalized to the corresponding value of TOV21G^LI^ cells. Data are expressed as the mean ± SEM. of n = 3 measurements. *, *P* < .05; **, *P* < .01; ***, *P* < .001. D, Wound healing of TOV21G^LI^ and TOV21G^HI^ cells was monitored and photographed at 0, 4, 8 and 12 h by using an optical microscope

**Figure 2:**
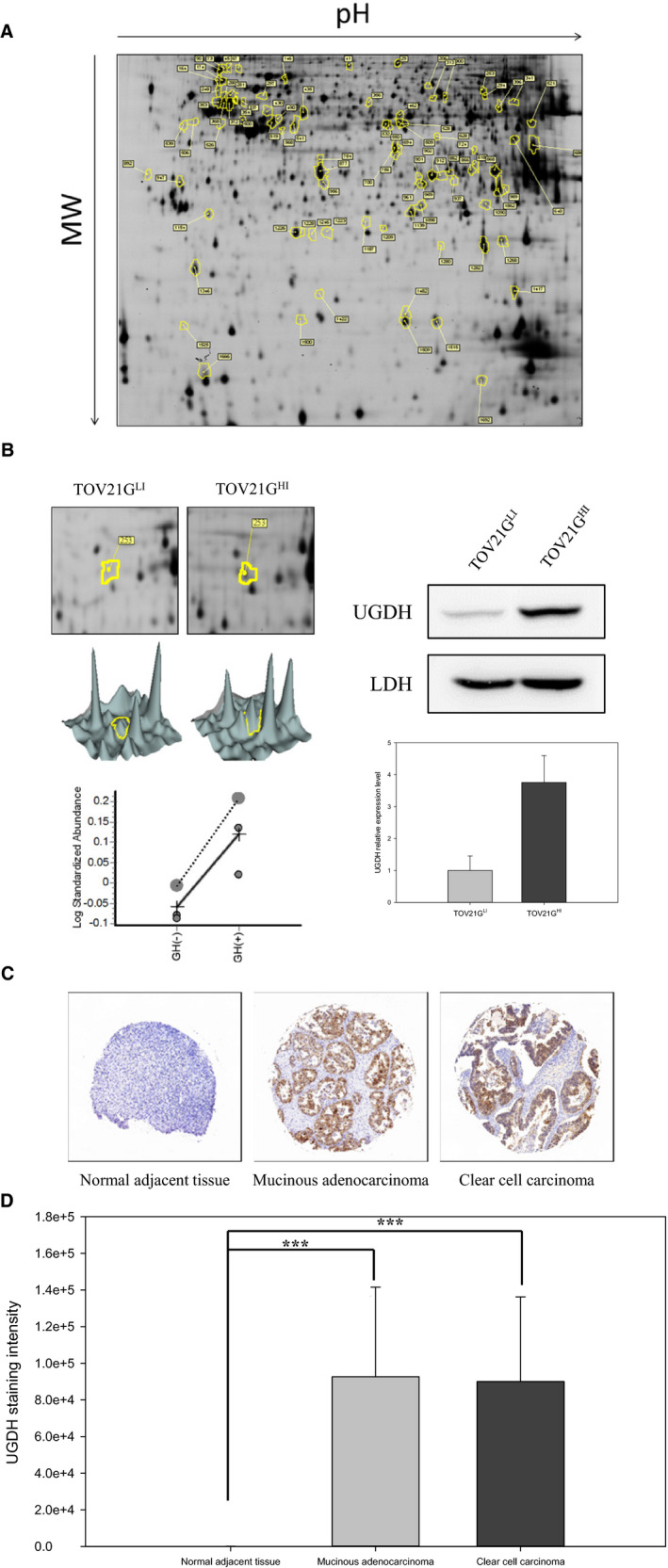
Proteomic analysis of metastasis‐related proteins and UGDH expression level in clinical tissue specimens of ovarian cancer. A, TOV21G^LI^ and TOV21G^HI^ cells were subjected to the 2D‐DIGE analysis. The results of 2D‐DIGE images were analysed by using DeCyder^™^ software; differentially expressed identified proteins with greater than 1.5‐fold or lower than −1.5‐fold differences are annotated with circles and spot numbers. B, Left panel: Up‐regulation of UGDH in TOV21G^HI^ cells was observed in the 2D image, 3D image, and statistical data from the analysis of DeCyder^™^ software. Right panel: Expression levels of UGDH in TOV21G^LI^ and TOV21G^HI^ cells were validated by immunoblotting. The relative expression level of UGDH was quantified by ImageQuant software and normalized with the expression of LDH. Data are represented as the mean ± SEM. **, *P* < .01 compared to the expression level of UGDH in TOV21G^LI^ cells. C, Expression level of UGDH in tissue microarray was monitored by immunohistochemical staining. The representative images show the expression level of UGDH in normal adjacent tissue, mucinous adenocarcinoma and clear cell carcinoma. D, UGDH expression intensities in normal adjacent tissue (n = 10), mucinous adenocarcinoma (n = 12) and clear cell carcinoma (n* = *5) were analysed by ImageJ software. The diagram represents the staining intensity of the clinical tissue samples. Data are represented as the mean ± SEM. ***, *P* < .001 compared to the malignant tumour group

To investigate the metastatic mechanism of ovarian cancer, we analysed the expression level of GH, a cancer‐specific marker,^18^ in TOV21G cells. We isolated two cell groups by BD FACSAria™ III cell sorter according to the expression level of GH. In our flow cytometry data, TOV21G^HI^ cells showed a higher expression level of GH compared to TOV21G^LI^ cells (Figure 1A). The immunofluorescence results revealed relatively higher expression level of GH in TOV21G^HI^ compared to in TOV21G^LI^ cells (Figure 1B). Moreover, TOV21G^HI^ cells exhibited significantly increased cell invasion and cell migratory abilities compared to TOV21G^LI^ cells (Figure 1C,D). Next, proteomic analysis was applied to elucidate the global protein changes between isogenic TOV21G^LI^ and TOV21G^HI^ cells. We detected 1863 proteins using DeCyder software and 217 proteins showed differential expression levels concerning the set values (average ratio ≥ 1.5‐fold, ≤‐1.5‐fold; *P* < .05) (Figure 2A). After MALDI‐TOF MS analysis and MASCOT database searching, the identified proteins were categorized according to their function and subcellular localization. Among all detected proteins, UGDH showed a high expression level in TOV21G^HI^ cells, based on 2D DIGE images and statistic data (Figure 2B). To further confirm our data of proteomic analysis, we performed immunoblotting to validate the expression level of UGDH between the TOV21G^LI^ and TOV21G^HI^ cell lines. The expression level of UGDH in TOV21G^HI^ cells was significantly higher than that in TOV21G^LI^ cells, suggesting that UGDH is overexpressed in a highly aggressive ovarian cancer cell line.

### 3.2 Expression of UGDH is correlated to aggressive types of ovarian cancer

In the cell line‐based study, we observed overexpression of UGDH in the highly aggressive ovarian cancer cell line. Subsequent analysis revealed overexpression of UGDH in highly invasive ovarian cancer tissue specimens. In this study, immunohistochemistry (IHC) was conducted to evaluate the expression level of UGDH in ovarian cancer tissue. All adjacent normal tissues (n = 10) showed weak expression of UGDH, whereas increased expression was observed in malignant tumours, including clear cell carcinoma and mucinous adenocarcinoma tissues. To evaluate the expression level of each tissue microarray specimen, we quantified the expression level of UGDH according to the staining intensity. IHC results indicated that UGDH was highly expressed in over 60% of clear cell carcinoma and mucinous adenocarcinoma tissue samples compared to in adjacent normal tissue (Figure 2D). These results support that overexpression of UGDH is not only related to tumour malignancy but also is a potential prognostic biomarker for ovarian carcinoma and mucinous adenocarcinoma.

### 3.3 Knockdown of UGDH reduces cell proliferation in ovarian cancer by prompting G1 phase arrest

**Figure 3:**
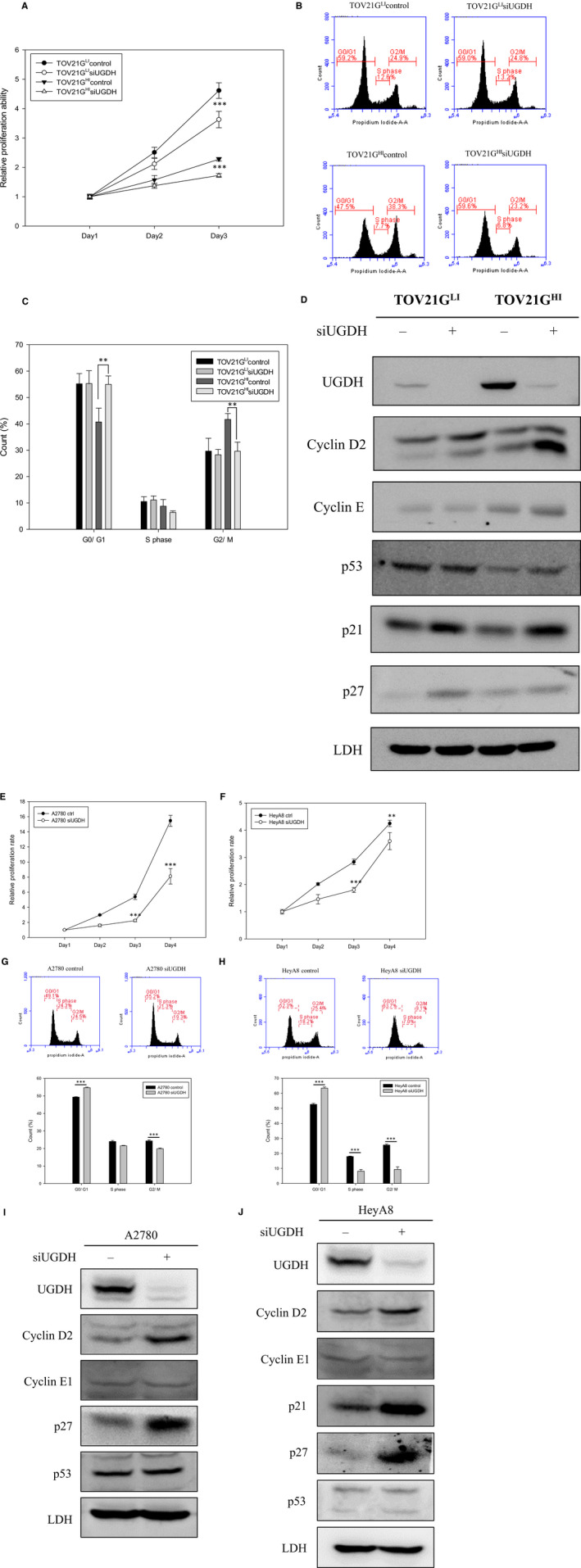
Deficiency of UGDH by siRNA inhibited cell proliferation and led to cell cycle arrest. A, Cell proliferation rates of TOV21G^LI^ and TOV21G^HI^ cells were monitored from days 1 to 3 after the treatment with siUGDH by MTT assay. The absorbance at days 2 and 3 was normalized to that at day 1. Data are presented as the mean ± SEM. ***, *P* < .001 compared to control ovarian cancer cells. B, Representative diagrams of cell cycle of control and siUGDH‐treated cells analysed by CFlow software. siUGDH‐treated TOV21G^LI^ and TOV21G^HI^ cells and control cells were stained with PI and applied to analyse DNA content by flow cytometry. The distribution of cell cycle phases (G_0_/G_1_, S, G_2_/M) is shown as indicated. C, Numbers of cells at the different cell cycle stages were statistically analysed and graphed. Data derived from three independent experiments are presented as the mean ± SEM. **, *P* < .01 compared to control cells. D, Expression levels of cell cycle regulatory proteins were monitored in response to siUGDH treatment. Immunoblotting was used to detect the expression level of cyclin D2, cyclin E, p53, p21 and p27 proteins in control and siUGDH‐treated TOV21G cells. Immunoblotting results of the indicated proteins were quantified by ImageJ and normalized to LDH. Cell proliferation assay via MTT assay was performed to analyse the effect of siUGDH on A2780 and HeyA8 cells (E), (F). siUGDH‐treated cells were monitored to assess the proliferation rates from days 1 to 4 by MTT assay. Data are presented as the mean ± SEM. **, *P* < .05; ***, *P* < .001 compared to control ovarian cancer cells. G, H, Representative plots of cell cycle analysis of A2780, HeyA8 and their siUGDH‐treated partners. I, J, Immunoblotting was performed to detect the expression levels of cyclin D2, cyclin E1, p21, p27 and p53 in A2780, HeyA8 and UGDH knockdown partners

Previous studies demonstrated that a deficiency of UGDH leads to reduced cell proliferation in colon cancer^12^ and glioblastoma.^13^ We hypothesized that knockdown of UGDH by short interfering RNA (siRNA) could similarly reduce cell proliferation in ovarian cancer. Three cell line models, TOV21G, A2780 and HeyA8, were used for cell proliferation analysis. Ovarian cancer cells were transfected with 50 nmol/L of UGDH siRNA for 4 hours and recovered in complete medium for at least 16 hours before analysis. Control cells and cells transfected with a specific UGDH‐targeting siRNA, siUGDH, were harvested and examined to determine the cell proliferation rate by MTT assay from starting at days 1‐3. As shown in Figure 3A, the relative proliferation rates were significantly lower in siUGDH‐transfected TOV21G^LI^ and TOV21G^HI^ cells than in control cells from days 2 to 3. Similar effects were observed in A2780 and HeyA8 cells following transfection with siUGDH (Figure 3E,F).

To further elucidate the regulatory role of UGDH in cell proliferation, we performed propidium iodide (PI) staining in combination with flow cytometry to analyse the cell cycle distribution in ovarian cancer cells. Flow cytometry showed that knockdown of UGDH increased the population of G_0/_G_1_ phase cells (47.5%–59.6%) and decreased the faction of G_2_ phase cells (38.3%–23.2%) in TOV21G^HI^‐siUGDH‐transfected cells compared to control cells (Figure 3B,C). Transfection of siUGDH also increased the number of G_0_/G_1_ phase cells (52.3%–63.7%) and decreased the portion of G_2_ phase cells (18.2%–7%) in HeyA8 cells compared to control cells (Figure 3H). In A2780 cells, siUGDH had the same effect on the cell cycle by arresting over 15% cells in G0/G1 phase (Figure 3G). We also examined the effects of UGDH siRNA on cell cycle regulators via immunoblotting. The expression levels of cyclin D2, p53, p21 and p27 were up‐regulated in response to treatment with siUGDH in TOV21G cells (Figure 3D). Both A2780 and HeyA8 cells showed elevated cyclin D2 and p27 after siRNA treatment (Figure 3I,J). Taken together, the deficiency of UGDH in TOV21G, A2780 and HeyA8 cells provoked up‐regulation of the cell cycle inhibitors p53, p21 and p27 and may lead to an increase in the population of G_0_/G_1_ phase cells and the decrease of cell numbers in G_2_ phase. Our results suggest that knockdown of UGDH suppressed the ovarian cancer proliferation rate and induced cell cycle arrest in G_0_/G_1_ phase by up‐regulating cell cycle inhibitory proteins.

### 3.4 Knockdown of UGDH through siRNA impairs wound healing and migration of ovarian cancer cells

**Figure 4:**
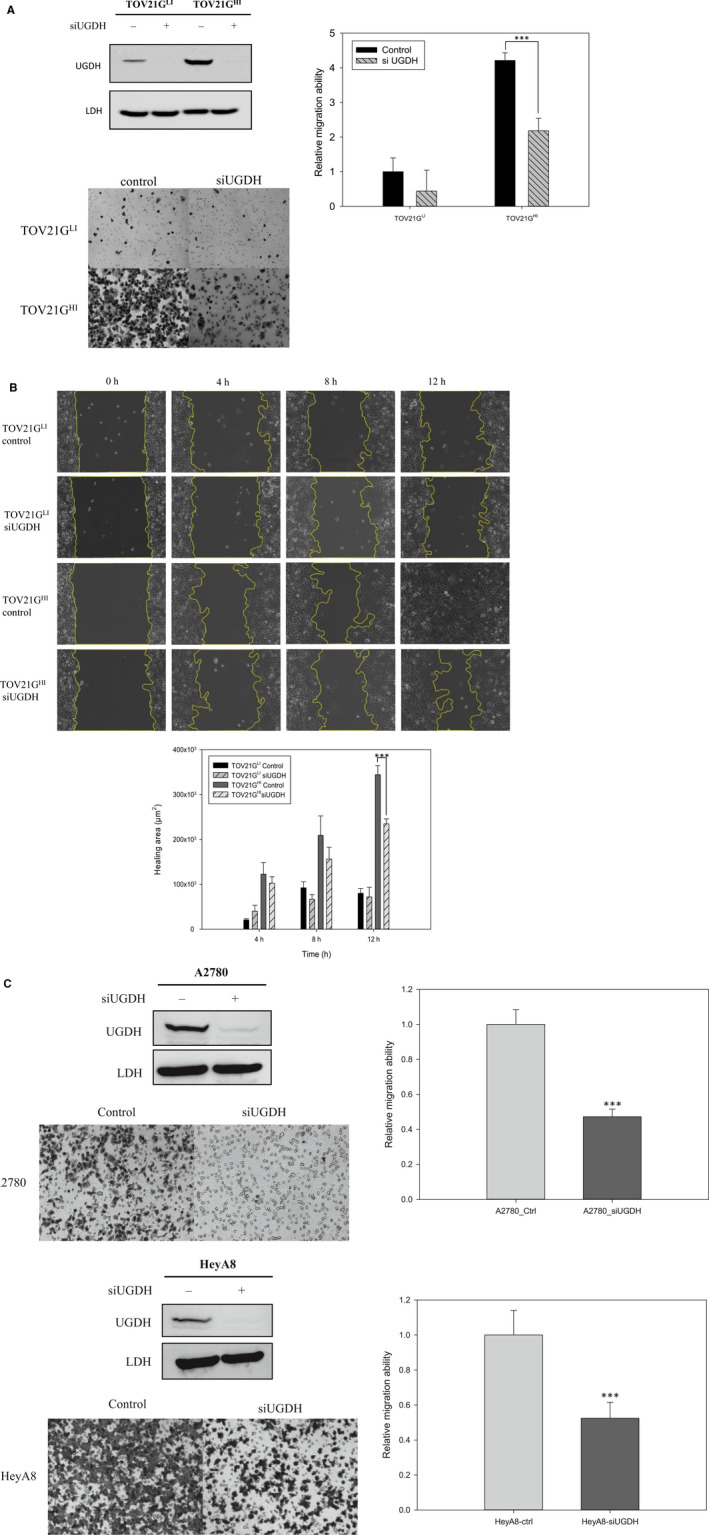
Deficiency of UGDH by siRNA decreased cell migration and wound healing ability in ovarian cancer cell lines. A, Left panel: Knockdown efficiency by siUGDH was measured by immunoblotting. UGDH siRNA efficiently decreases ~90% of the protein level in both TOV21GLI and TOV21GHI cells. Middle: Representative images of the migrated cells at 10× magnification using an optical microscope. Right panel: transwell migration assay was performed to monitor the effect of siUGDH on the cell migration ability. After seeding the cells in the transwell insert for 18 h, migrated cells were fixed and stained with crystal violet for quantification. Absorbance values were normalized to the value of TOV21G^LI^ control cells. Data are represented as the mean ± SEM. ***, *P* < .001 compared to control ovarian cancer cells. B, Wound healing assay showed the same trend as the transwell assay. After treatment with siRNA, the wound healing of ovarian cancer cells was monitored and photographed at 0, 4, 8 and 12 h by an using optical microscope. The healing areas at certain times were quantified by AxioVision 4.8 software. The amounts of healing area were normalized to the amount at 0 h. Data are represented as the mean ± SEM. ***, *P* < .001 compared to control ovarian cancer cells. C, Knockdown efficiency of siUGDH in A2780 and HeyA8 cells was monitored by immunoblotting. Transwell migration assay was applied to monitor the effects of siUGDH in A2780 and HeyA8 cells

We next focused on the role of UGDH in cell wound healing and cell migration in ovarian cancer by wound healing assay (scratch assay) and transwell migration assay. UGDH was knocked by siRNA in three ovarian cancer cell models, TOV21G, A2780 and HeyA8 cells. The cell migratory ability was significantly decreased when TOV21G^HI^ cells were transfected with siUGDH (Figure 4A). Transwell migration assay revealed that knockdown of UGDH significantly decreased the migration ability of TOV21G^HI^ cells compared to control cells. Similar effects were observed in A2780 and HeyA8 cells (Figure 4C). Our data demonstrate that knockdown of UGDH attenuated the migratory ability of ovarian cancer cells. The wound healing areas in TOV21G^HI^ cells transfected with siUGDH were significantly impaired compared to in TOV21G^HI^ control cells at 12 hours (Figure 4C), whereas siUGDH did not alter the wound healing ability in TOV21G^LI^ cells. Based on these findings, silencing of UGDH reduced the migration ability in TOV21G^HI^, A2780 and HeyA8 cells.

### 3.5 Knockdown of UGDH decreased ovarian cancer tumour growth in xenograft model

**Figure 5:**
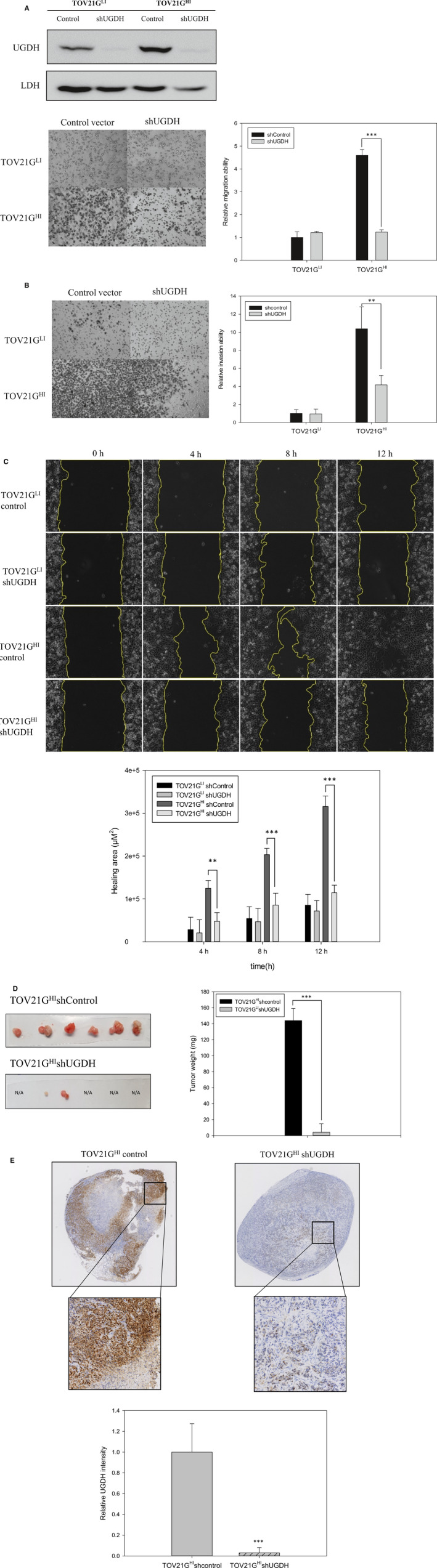
Deficiency of UGDH by shRNA decreased tumour growth in a xenograft model in vivo. Knockdown efficiency of shRNA was measured by immunoblotting. The results showed that UGDH shRNA decreased the protein expression level of UGDH in TOV21G^LI^ and TOV21G^HI^ cell lines by more than 80%. Transwell migration assay and transwell Matrigel invasion assay were performed to examine the effects of shUGDH in TOV21G cells. A, B, Migrated and invaded cells in transwell assays are shown in representative images. The values of relative metastatic ability were normalized to the cells transduced with an empty vector. Data derived from three independent experiments are presented as the mean ± SEM. **, *P* < .01; ***, *P < *.001 compared to cells transduced with empty vector. C, Wound healing of TOV21G^LI^ control, TOV21G^LI^ shUGDH, TOV21G^HI^ control and TOV21G^HI^‐shUGDH cell lines were observed and photographed at 0, 4, 8 and 12 h by optical microscopy. The healing areas at certain times were quantified with AxioVision 4.8 software. Values of the healing area were normalized to that of each experimental group at 0 h. Data are represented as the mean ± SEM. ***, *P* < .001 compared to control ovarian cancer cells. D, Tumour growth of TOV21G^HI^ control (n = 6 mice) and TOV21G^HI^‐shUGDH (n = 6 mice) in the xenograft model via subcutaneous injection. The tumour weights were measured, and the results were statistically analysed. Data are represented as the mean ± SEM. ***, *P* < .001 compared to the cells transduced with empty vector group. E, Immunohistochemical images represent nuclear staining (blue colour) and the expression level of UGDH (brown colour). Bottom: Zoom‐in images of indicated areas. The UGDH intensity of TOV21G^HI^ control and TOV21G^HI^‐shUGDH tumour tissue was detected and quantified by ImageJ software. Data are presented as the mean ± SEM. ***, *P* < .001 compared to the TOV21G^HI^ control group

The results revealed that knockdown of UGDH decreased ovarian cancer migration, wound healing ability and cell proliferation. Thus, we hypothesized that a deficiency in UGDH would influence the tumour growth rate in a xenograft model. We established UGDH‐stable knockdown cell lines by introducing control empty vector and UGDH short hairpin RNA (shRNA) in both TOV21G^LI^ and TOV21G^HI^ cells. Immunoblot analysis showed ~90% inhibition of UGDH in both TOV21G^LI^ and TOV21G^HI^ cells (Figure 5A left) by shRNA. To confirm the effect mentioned above in siUGDH‐treated assays, we performed transwell migration and wound healing assays with UGDH‐stable knockdown cells and control cells. As shown in Figure 5A, knockdown of UGDH by shRNA significantly impaired the migratory ability of TOV21G^HI^ cells. The wound healing assay also showed that the healing ability was reduced in shRNA‐mediated UGDH knockdown TOV21G^HI^ cells compared to in control cells (Figure 5C). We also performed a Matrigel invasion assay to monitor the effect of UGDH on cell invasive ability. The results showed that knockdown of UGDH by shRNA significantly decreased the cell invasive ability of TOV21G^HI^ cells compared to cells transduced with the empty vector (Figure 5B).

To examine the effect of UGDH silencing on ovarian tumour growth in vivo, we employed TOV21G^HI^ cells to perform xenograft transplantation of immunodeficient mice in vivo. Subcutaneous injections were performed on the hindlimbs of mice by implanting TOV21G^HI^ cells that stably expressed either control empty vector or UGDH shRNA. The animals were killed at 28 days after injection. Our results demonstrated that the shRNA‐mediated deficiency of UGDH significantly decreased tumour growth. Furthermore, the tumour weights in mice implanted with TOV21G^HI^ cells expressing UGDH shRNA were sixfold lighter than those in mice implanted with TOV21G^HI^ cells expressing the empty vector (Figure 5D). IHC staining of the tumour sections confirmed the lower expression level of UGDH in the UGDH knockdown group compared to in the control group (Figure 5E), indicating the long‐term inhibition via shRNA in our injected cells. Collectively, our results demonstrated that knockdown of UGDH not only attenuated ovarian cancer migration and invasion abilities in vitro but also decreased ovarian tumour growth in a xenograft model in vivo.

### 3.6 Knockdown of UGDH decreased gh expression level and impaired metastatic ability of ovarian cancer cells by inhibiting mapk signalling pathway, F‐actin polymerization and EMT

**Figure 6:**
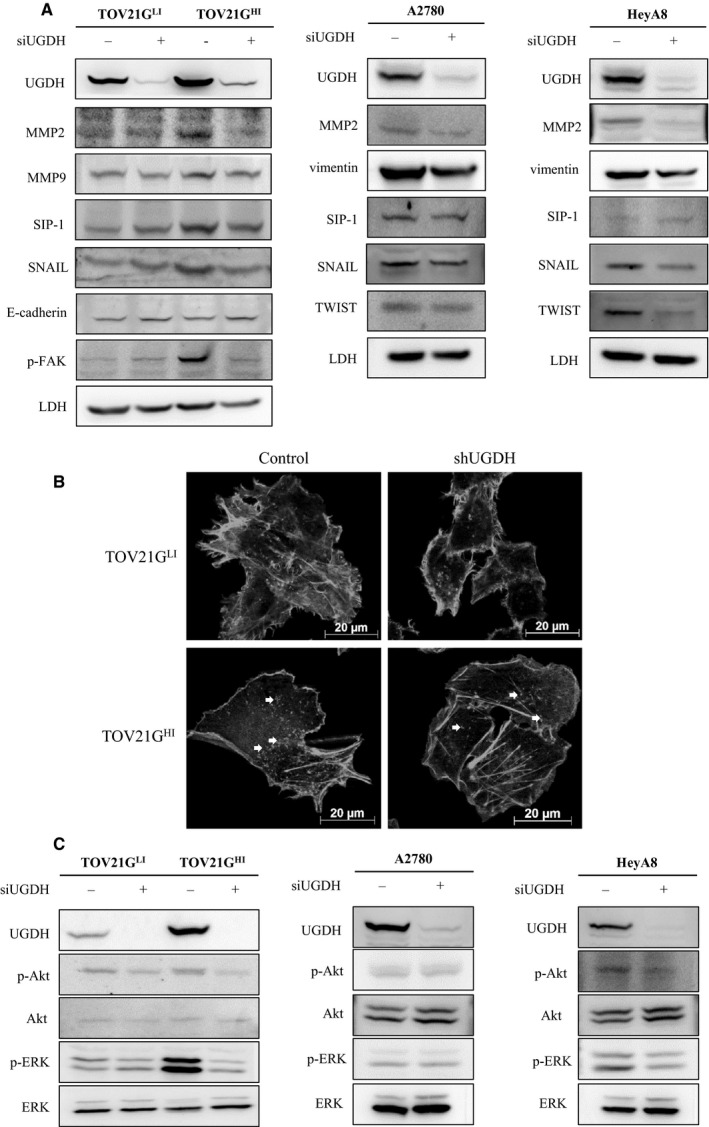
Knockdown of UGDH modulated F‐actin reorganization, MAPK signalling pathway, EMT‐related factors and expression level of Globo H. A, Immunofluorescence representative images of F‐actin expression status in TOV21G control and TOV21G‐shUGDH cell lines. F‐actin of cell lines was stained with rhodamine‐labelled phalloidin and photographed using a fluorescence microscope. The white arrows indicate actin dots in TOV21G^HI^ control and TOV21G^HI^‐shUGDH cell lines. B, Immunoblotting analysis of MAPK pathway factors (ERK/p‐ERK, AKT/p‐AKT), EMT‐related factors (SIP‐1, SNAIL and E‐cadherin), phosphorylated focal adhesion kinase (p‐FAK), MMP2 and MMP9 in TOV21G control and TOV21G‐shUGDH cell lines. The protein expression values were quantified with ImageJ, and the relative expression level of each protein was calculated by normalizing to the protein expression value of TOV21G^LI^ control

To understand the regulatory role of UGDH in ovarian cancer metastasis, we focused on monitoring the molecular pathway of UGDH knockdown in TOV21G cells. We investigated the expression level of the EMT markers in UGDH knockdown cell lines. EMT is recognized to have a critical role in cancer metastasis. SIP‐1, SNAIL and TWIST were reported as transcription factors with major regulatory roles in cancer metastasis.^19^ Our immunoblot analysis showed that SIP‐1 and SNAIL were up‐regulated in TOV21G^HI^ cells, representing enhanced EMT progression. Additionally, SIP‐1 and SNAIL were down‐regulated, and E‐cadherin was up‐regulated in an siUGDH‐treated TOV21G^HI^ cell line compared to in the control cell line. In A2780 cells, the expression levels of vimentin, SIP‐1, SNAIL and TWIST were reduced upon treatment with siUGDH. Similar results were observed in siUGDH‐treated HeyA8 cells, but the level of SIP‐1 showed no noticeable change after siRNA treatment. Matrix metalloprotease protein 2 (MMP2), serving as a cancer metastasis promoting factor,^20^ was also down‐regulated in both UGDH‐silencing TOV21G, A2780 and HeyA8 cells. Interestingly, knockdown of UGDH also decreased the phosphorylation of focal adhesion kinase in TOV21G^HI^ cells (Figure 6A). Actin reorganization a critical factors modulating cell motility. In migrating cells, actin undergoes dynamic assembly and disassembly, which regulates focal adhesion and contractile filament, inducing thereby cell migration.^21^, ^22^ Therefore, we performed F‐actin staining by phalloidin in TOV21G cell lines to monitor the status of actin in response to the knockdown of UGDH. The number of actin dots in TOV21G^HI^ cells transduced with empty vector was notably higher than in TOV21G^LI^ cells, suggesting that TOV21G^HI^ control cells were relatively more motile (Figure 6B). Actin dots were decreased in TOV21G^HI^‐expressing UGDH shRNA cells, supporting the relatively lower migratory ability of these cells. Furthermore, several studies mentioned that transforming growth factor‐β and interleukin‐1β can induce the expression of UGDH via the MAPK pathway.^23^, ^24^ In our study, silencing of UGDH inhibited the phosphorylation of ERK, indicating bi‐directional regulation between the UGDH and MAPK pathways.

## 4 DISCUSSION

Cancer metastasis not only accounts for most cancer‐related death but also is a major clinical obstacle to cancer therapy. In ovarian cancer, most patients have regional or distant metastasis at the time of diagnosis.^25^, ^26^ Therefore, identification of new diagnostic and therapeutic targets is an urgent issue in ovarian cancer treatment. To discover new therapeutic targets, we generated the highly invasive ovarian cancer cell line TOV21G^HI^ and low‐invasive line TOV21G^LI^ based on the expression level of GH, a cancer‐specific marker. In previous reports, GH was detected in a variety of epithelial cell cancers, including breast, colon, gastric, lung, ovarian and prostate cancers using the anti‐Globo H antibody Mbr1.^27^, ^28^, ^29^

Additionally, Cheng et al^9^ demonstrated that highly GH expressing breast cancer cells showed faster growth rate and greater blood vessel density than low GH expressing cells in a xenograft model in vivo. We observed enhanced wound healing and invasive abilities in highly GH expressing ovarian cancer cells, TOV21G^HI^. Thus, the expression of GH not only serves as a cancer‐specific marker but is also responsible for the high invasiveness of cancer cells. To further examine the detailed mechanism of ovarian cancer metastasis, we performed 2D‐DIGE in combination with MALDI‐TOF MS to investigate the potential proteins involved in ovarian cancer metastasis. Among the identified proteins, UGDH was found to be elevated in TOV21G^HI^ cells.

UDP‐glucose dehydrogenase was reported to induce prostate cancer progression and can serve as a potential biomarker of prostate cancer.^30^ In a report from The Human Protein Atlas, overexpressed UGDH is associated with poor survival rates in renal cancer.^31^ Oyinlade et al^13^ also showed that high expression of UGDH in glioblastoma leads to poor survival rates. Our proteomics data showed that UGDH is overexpressed in the highly invasive cell line TOV21G^HI^. Tissue microarray analysis showed that UGDH is highly expressed in clear cell carcinoma and mucinous carcinoma tissue samples, but not in normal adjacent tissue. Clear cell carcinoma and mucinous carcinoma belong to Type I ovarian cancer and together account for ~ 10% of ovarian cancer, whereas serous carcinoma accounts for ~90% of ovarian cancer.^32^ Despite the low occurrence, both show poor prognosis and are more lethal compared to serous carcinoma at stages III and IV.^32^, ^33^ In addition, in vitro analysis of ovarian cancer cell lines showed that non‐serous ovarian cancer cell lines had more aggressive migration and invasion ability.^34^ These results suggest that UGDH is a potential biomarker for clear cell carcinoma and mucinous cell carcinoma and that the expression of UGDH may be related to a poorer prognosis in ovarian cancer.

We found that knockdown of UGDH leads to the decrease in the cell proliferation rate in TOV21G, A2780 and HeyA8 cells. Moreover, G_1_ phase cell cycle arrest was observed in UGDH knockdown cells. These results are consistent with previous reports in glioblastoma cells in which knockdown of UGDH by shRNA decreased cell proliferation and displayed a delay in G_0_/G_1_ to S phase transition.^13^ Our further studies of the expression level of cell cycle regulators showed that p21 and p27 were up‐regulated in UGDH knockdown cells. A recent report suggested that expression of p21 and p27 lead to cell cycle arrest in G_0_/G_1_ phase, ultimately resulting in cell death in lung cancer.^35^ Our results also showed that p21 and p27 were elevated in UGDH knockdown cells, suggesting that loss of UGDH leads to increases in cell cycle inhibitors, which cause cell cycle arrest in G_0_/G_1_ phase. Our immunoblot analysis revealed that cyclin D2 was elevated in UGDH knockdown ovarian cancer cells. D‐Type cyclins (cyclin D1, cyclin D2 and cyclin D3) play crucial roles in the cell cycle machinery. The cyclin D family is associated with partner cyclin‐dependent kinases, CDK‐4 and CDK‐6, and the complexes promote the phosphorylation of retinoblastoma protein, resulting in the release of E2F transcription factor and allowing the cell cycle to shift from G_1_ to S phase. We found that cyclin D2 was elevated in UGDH‐deficient G_0_/G_1_ arrest cells, which is contentious to our current understanding. However, several studies reported that cyclin D2 plays distinct roles in cell cycle regulation and cancer progression. Reduced expression of cyclin D2 was observed in breast cancer, non‐small cell lung cancer, pancreatic cancer and prostate cancer, indicating that cyclin D2 may act as a tumour suppressor in these cancers.^36^, ^37^, ^38^, ^39^ Loss of CCD2 gene, which encodes cyclin D2, was also observed in renal cell cancer tissues and is correlated with aberrant methylation.^40^ Moreover, Meyyappan and colleagues reported that overexpression of cyclin D2 in human fibroblast cells leads to inhibition of cell cycle progression and DNA synthesis.^41^ These findings suggest that cyclin D2 plays a tumour suppressor role in cancer cells by negatively regulating cell cycle progression, which is consistent with our results in UGDH knockdown cell lines. Collectively, our data suggest that knockdown of UGDH causes cell cycle arrest by elevating the cell cycle regulators p21, p27 and cyclin D2.

**Figure 7:**
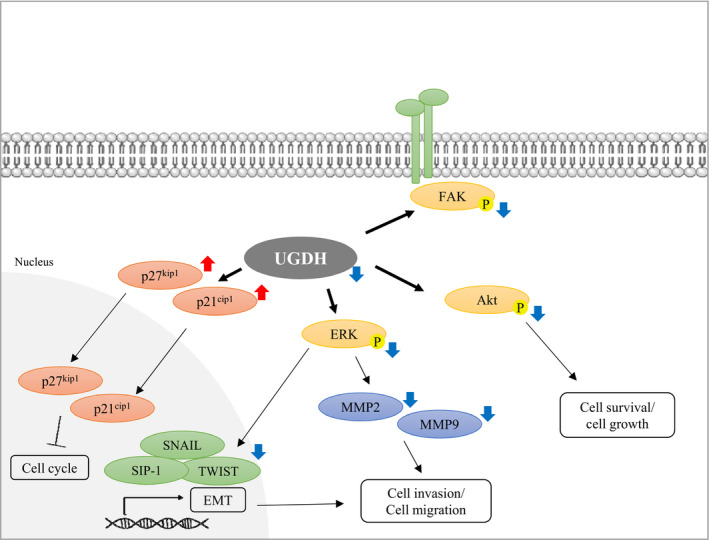
Hypothetical model representing the effect of UGDH knockdown in metastatic ovarian cancer cells. In UGDH knockdown cells, inactivation of ERK leads to down‐regulation of SIP‐1, SNAIL, TWIST, MMP 2 and MMP 9, which attenuate EMT, cell invasion and cell migration. Knockdown of UGDH causes elevation of p21^cip1^ and p27^kip1^ and inactivation of Akt, which inhibit cell cycle and cell growth

ERK, together with p38 and c‐Jun N‐terminal kinase/stress‐activated protein kinase, is three major MAPKs in mammalian cells. These MAPKs are associated with cell motility, invasion, survival, cell proliferation and morphogenesis.^42^ In our study, knockdown of UGDH decreased the phosphorylation of ERK in ovarian cancer cells, suggesting that UGDH is upstream of ERK in the signalling cascade. Thus, loss of UGDH expression contributes to the inhibition of ovarian cancer cell proliferation and cell invasion. In addition, numerous studies mentioned that MMP‐2 and MMP‐9, which are both regulated by the ERK/MAPK pathway, participate in cancer progression, including tumour growth, angiogenesis, cell invasion and migration.^42^, ^43^ Furthermore, overexpression of MMP‐2 and MMP‐9 was observed in ovarian cancer,^44^ and knockdown of both MMP proteins diminished the cell invasiveness of ovarian cancer.^45^, ^46^ Our immunoblot analysis revealed decreased expression levels of MMP‐2 and MMP‐9 in UGDH‐silenced ovarian cancer cells, which is consistent with previous reports. There is growing evidence that the ERK/MAPK pathway participates in the regulation of EMT‐related factors such as E‐cadherin, vimentin, SNAIL, SIP‐1 and TWIST.^47^, ^48^, ^49^ Our data demonstrated that UGDH overexpressing TOV21G^HI^ cells express higher levels of phosphorylated‐ERK, MMP‐2, SIP‐1 and SNAIL, whereas knockdown of UGDH leads to decreases in these markers, indicating that UGDH is involved in regulating ERK, MMP‐2 and EMT markers in ovarian cancer. We also revealed that knockdown of UGDH inhibits EMT, possibly through the ERK/MAPK pathway (Figure 7).

UDP‐glucose dehydrogenase serves as a metabolic enzyme and converts UDP‐glucose to UDP‐glucuronic acid, which is an initial step in the synthesis of GAGs.^50^ GAGs including chondroitin sulphate, dermatan sulphate, heparin sulphate and hyaluronan are the major components of extracellular matrix that participate in regulating several cancer cell behaviours, such as invasion, migration and angiogenesis.^51^ Chondroitin sulphate binds to fibroblast growth factor‐2 and vascular endothelial growth factor and forms complexes with their receptors, which enhances the signalling cascade and promotes cancer progression.^51^, ^52^ Moreover, heparin sulphate can interact with fibroblast growth factor‐2 (FGF‐2) and trigger cell proliferation and EMT by inducing FGF‐2 signalling.^53^, ^54^ In the current study, we observed overexpression of UGDH in highly invasive ovarian cancer cells, and siRNA‐mediated knockdown of UGDH reduced the metastatic abilities of TOV21G, A2780 and HeyA8 cell lines. We predict that knockdown of UGDH decreased the levels of GAGs in ovarian cancer and therefore affected the ovarian cancer metastatic ability. Previous studies revealed decreased expression levels of GAGs and hyaluronic acid in UGDH‐silenced colorectal carcinoma ^12^ and glioblastoma.^13^ Further studies are needed to demonstrate the effect of UGDH on the levels of GAGs in ovarian cancer. Additionally, we found that the expression level of GH was reduced in both UGDH shRNA transduced TOV21G^LI^ and TOV21G^HI^ cells, suggesting that knockdown of UGDH not only affects the amounts of GAGs but also reduces the expression of Globo H (Figure S1). Furthermore, previous report mentioned that quercetin, a polyphenol, had inhibitory effect on the enzyme activity of UGDH.^55^ To comprehensively understand the inhibitory mechanism of quercetin on UGDH, we used quercetin for analysing the effects of quercetin on cell migration and cell invasion ability. Our results demonstrated that quercetin inhibited the cell survival of TOV21G cells (Figure S2A). Moreover, treatment of quercetin also inhibited the migration and invasive ability of TOV21G cells (Figure S2B,C).

In conclusion, we demonstrated that UGDH is related to high invasiveness in ovarian cancer and provided evidence supporting that UGDH participates in cancer migration, invasion and cell proliferation in ovarian cancer. Collectively, UGDH can serve as a prognostic marker and a potential therapeutic target for restricting ovarian cancer metastasis. Future studies are likely to develop small molecular inhibitors specific for UGDH and to elucidate the relation between UGDH‐related glycan and metastasis.

## CONFLICT OF INTEREST

The authors confirm that there are no conflicts of interest.

## AUTHOR CONTRIBUTIONS

**Li‐Hsun Lin:** Conceptualization (equal); Data curation (equal); Formal analysis (equal); Funding acquisition (equal); Investigation (equal); Methodology (equal); Project administration (equal); Writing‐original draft (equal); Writing‐review & editing (equal). **Hsiu‐Chuan Chou:** Conceptualization (equal); Data curation (equal); Formal analysis (equal); Funding acquisition (equal); Investigation (equal); Methodology (equal); Project administration (equal). **Shing‐Jyh Chang:** Conceptualization (equal); Data curation (equal); Formal analysis (equal); Resources (equal); Software (equal); Supervision (equal). **En‐Chi Liao:** Data curation (equal); Formal analysis (equal); Resources (equal); Software (equal). **Yi‐Ting Tsai:** Data curation (equal); Formal analysis (equal); Resources (equal); Software (equal). **Yu‐Shan Wei:** Conceptualization (equal); Data curation (equal); Formal analysis (equal); Resources (equal); Software (equal). **Hsin‐Yi Chen:** Data curation (equal). **Meng‐Wei Lin:** Conceptualization (equal); Data curation (equal); Formal analysis (equal); Resources (equal); Software (equal). **Yi‐Shiuan Wang:** Data curation (equal); Formal analysis (equal); Resources (equal); Software (equal). **Yu‐An Chien:** Data curation (equal); Formal analysis (equal); Software (equal). **Xin‐Ru Yu:** Data curation (equal); Formal analysis (equal); Software (equal). **Hong‐Lin Chan:** Conceptualization (equal); Data curation (equal); Formal analysis (equal); Funding acquisition (equal); Investigation (lead); Methodology (equal); Project administration (lead); Supervision (lead).

## Supporting information

Supplementary Material

## Data Availability

The authors confirm that the data supporting the findings of this study are available within the article and its Supporting Information.
