# Supplementary material for: Targeting UDP‐glucose dehydrogenase inhibits ovarian cancer growth and metastasis
Source: J Cell Mol Med. 2020 Sep 7;24(20):11883–902. doi: 10.1111/jcmm.15808 (PMC7578908; doi:10.1111/jcmm.15808)
Supplement: Supplementary file 1 — Supplementary Material [file JCMM-24-11883-s001.docx]

**Figure S1.**GH expression level of TOV21G cells transduced with either control empty vector or UGDH shRNA. GH expression levels in TOV21G cells were detected by Mbr-1 antibody and FITC-conjugated secondary antibody using flow cytometry. Representative plots display the GH intensity of each cell line.


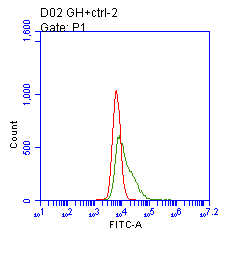

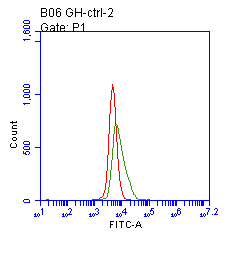


TOV21^HI^shcontrol

TOV21^HI^shUGDH

TOV21^LI^shcontrol

TOV21^LI^shUGDH

***

***

**Figure S2.** The effects of quercetin treatment on ovarian cancer migration and invasion. (A) TOV21G^LI^ (top) and TOV21G^HI^ (bottom) cell were incubated with different concentration of quercetin for 24 h. The effect of quercetin on cell viability was monitored by MTT assay. TOV21G^LI^ and TOV21G^HI^ cells were treated with 150μM of quercetin for 24 h. (B) The cell migration ability was measured by transwell migration assay. Cells were seeded into the upper insert of transwell for 8 h and the migrated cells were stained with crystal violet for microscope imaging. After crystal violet staining, cells were treated with dissolved buffer for following quantification of crystal violet intensity (C) The invasion ability was measured by matrigel pre-coated transwell assay. Cells were seeded into the upper chamber in transwell for 16 h. The invaded cells were stained with crystal violet followed by the quantification of intensity.

(A)

(B)


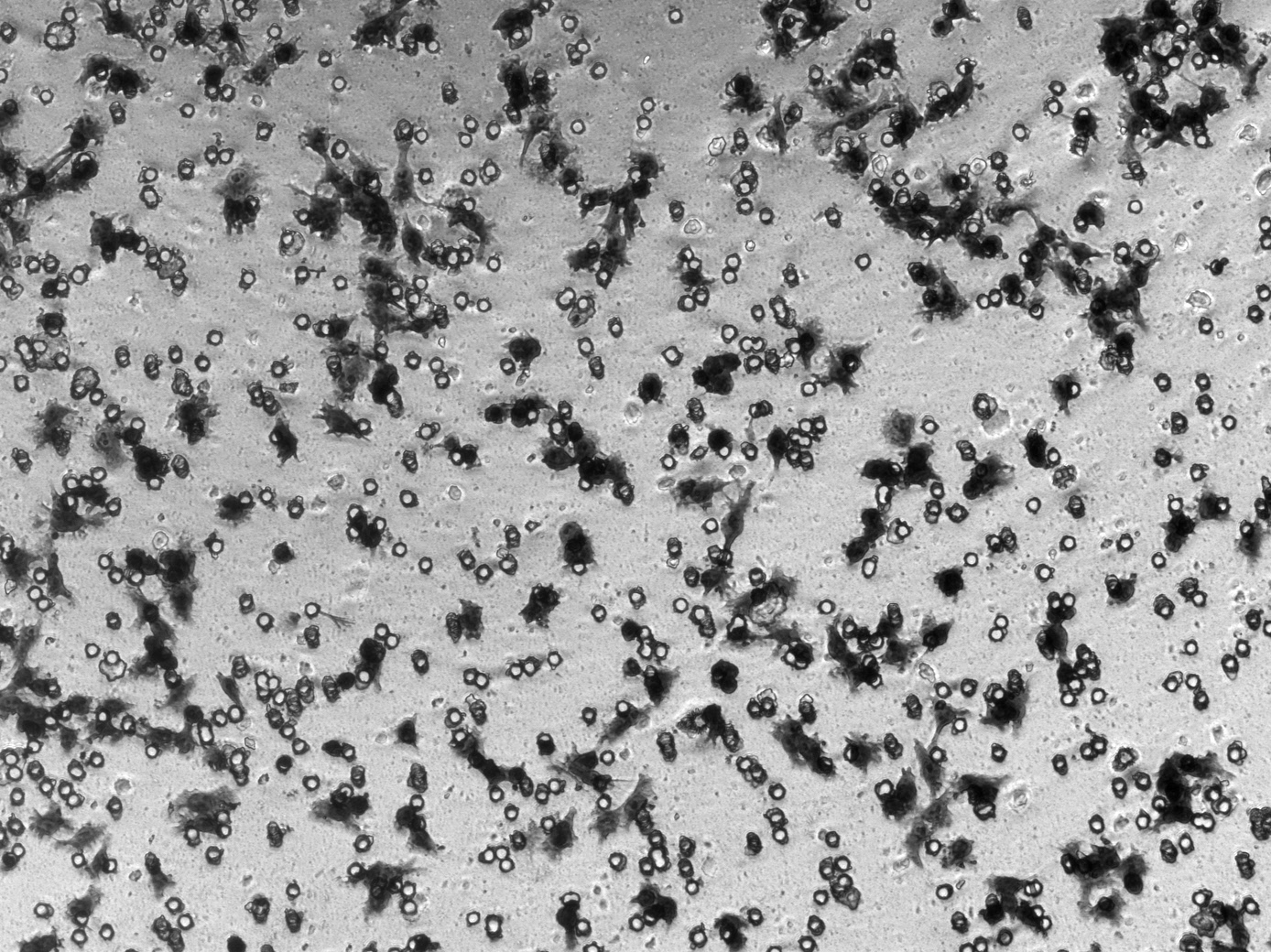

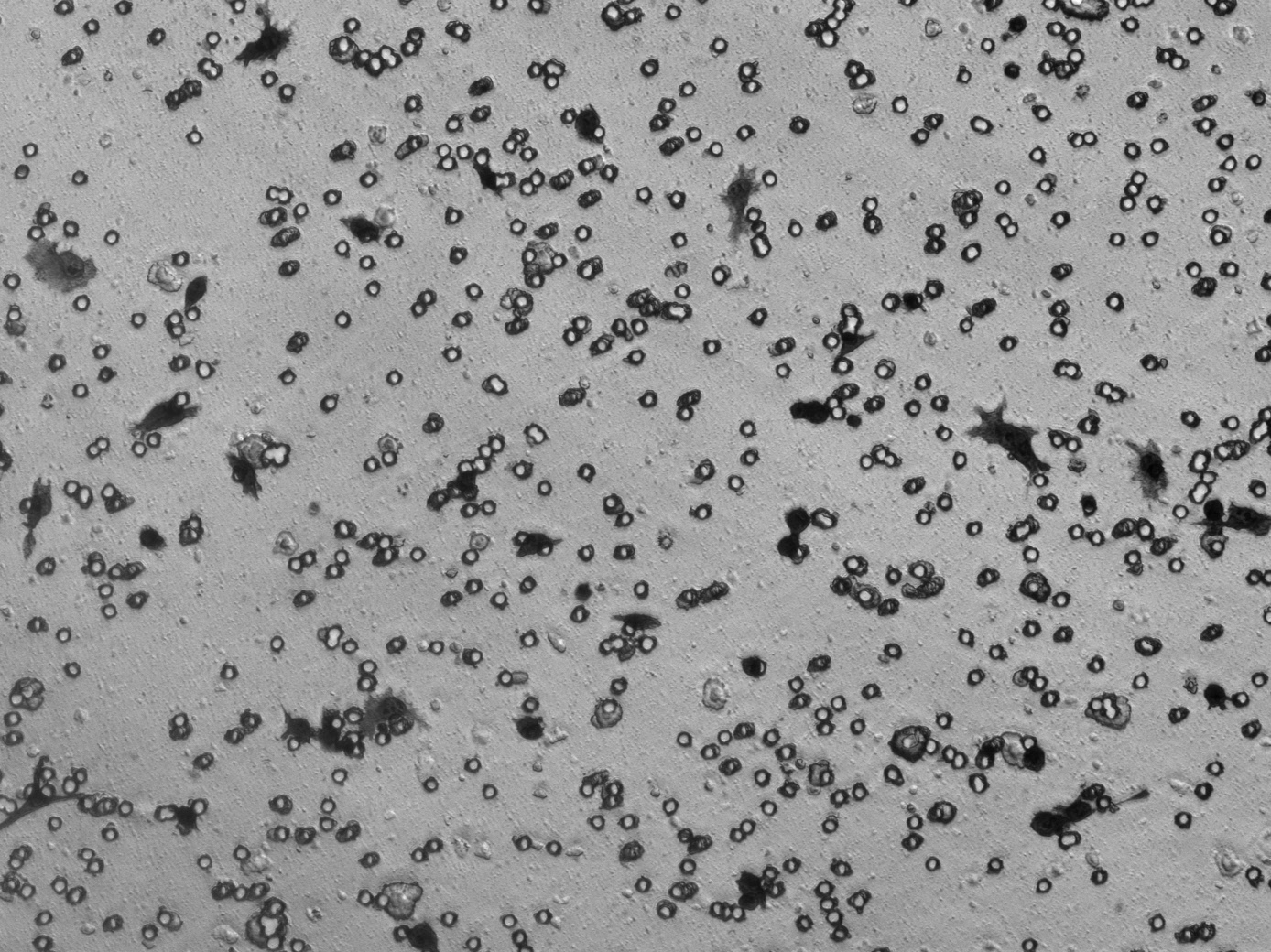

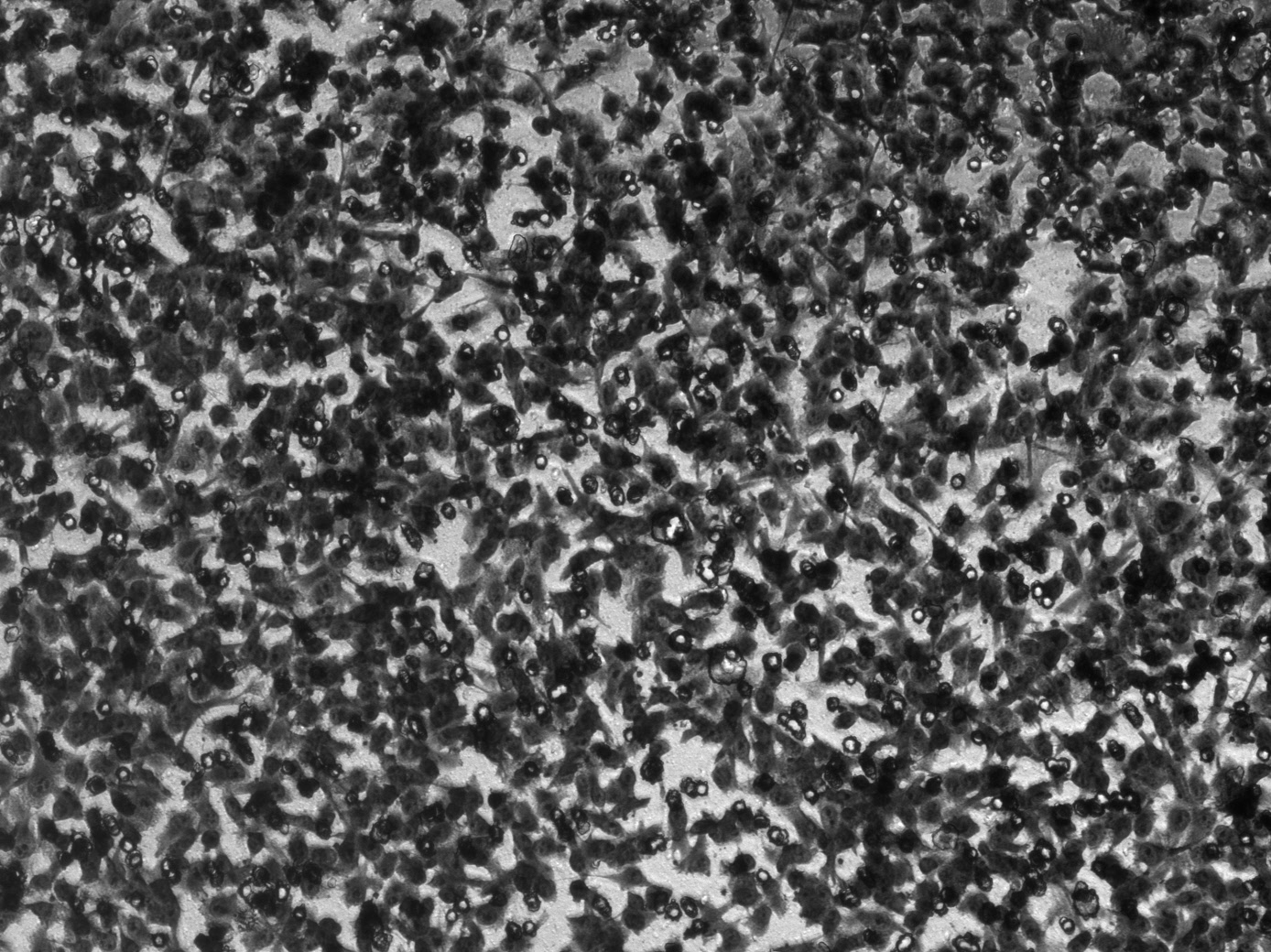

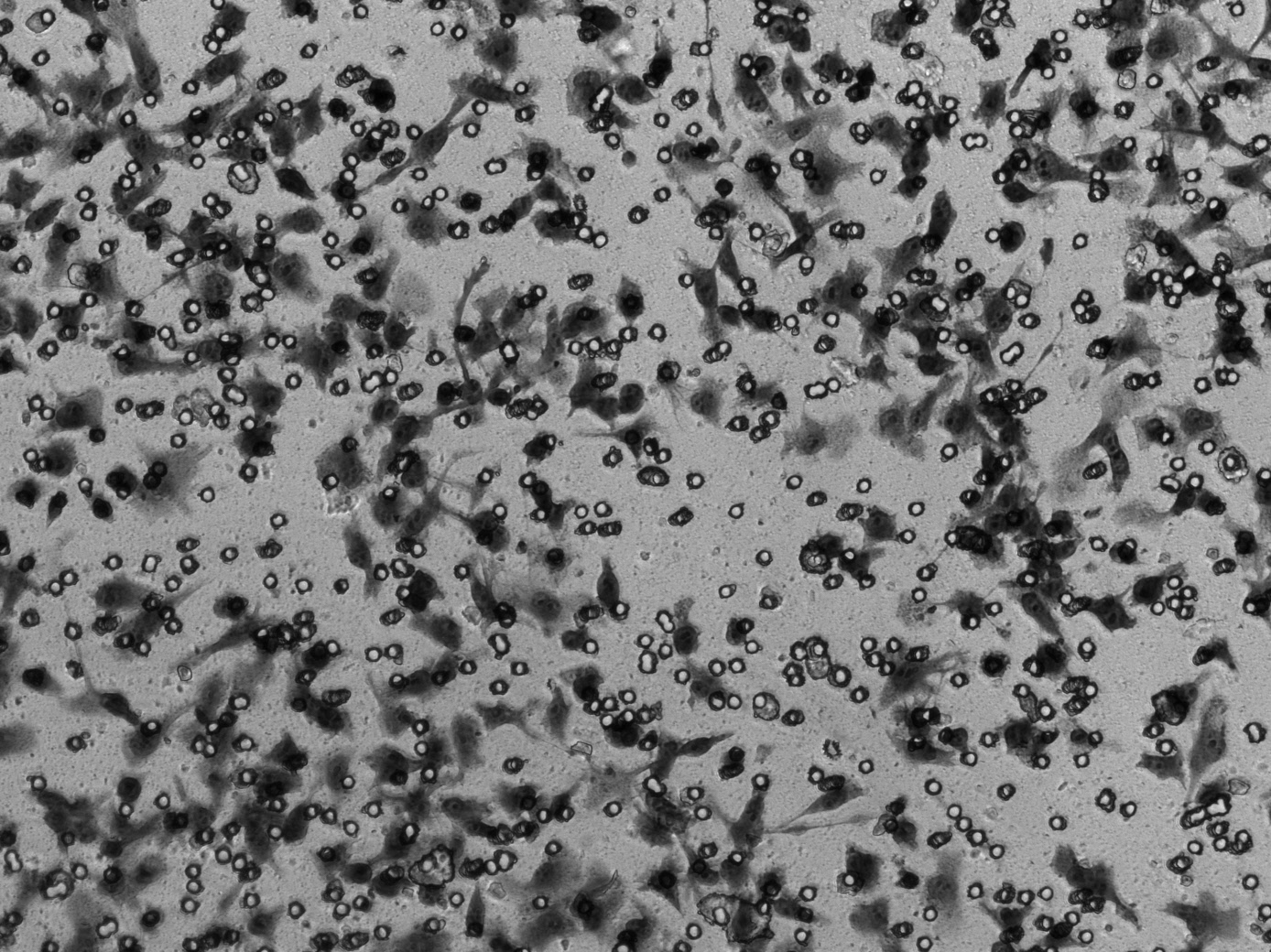


control

quercetin

TOV21G^LI^

TOV21G^HI^

(C)

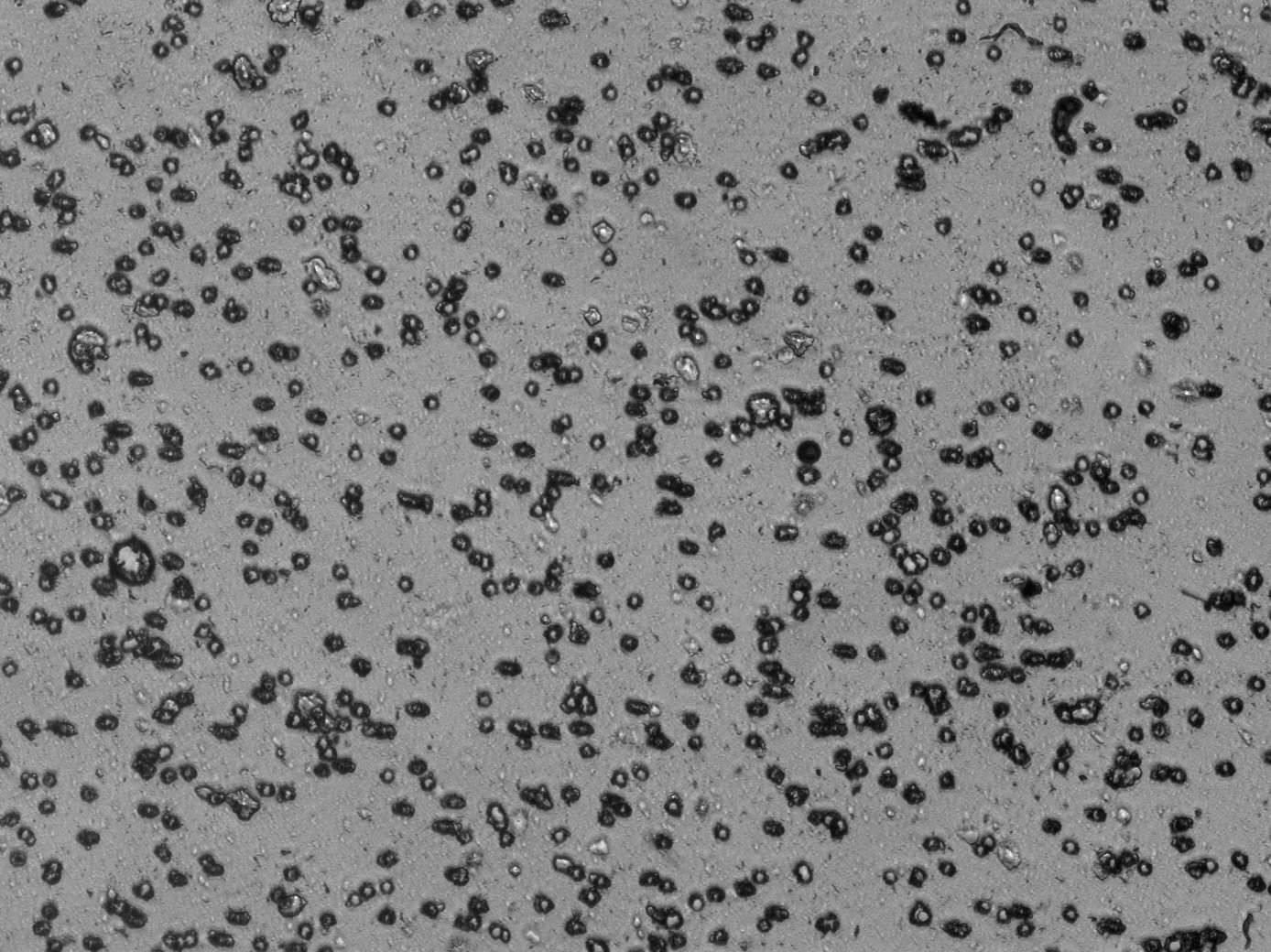

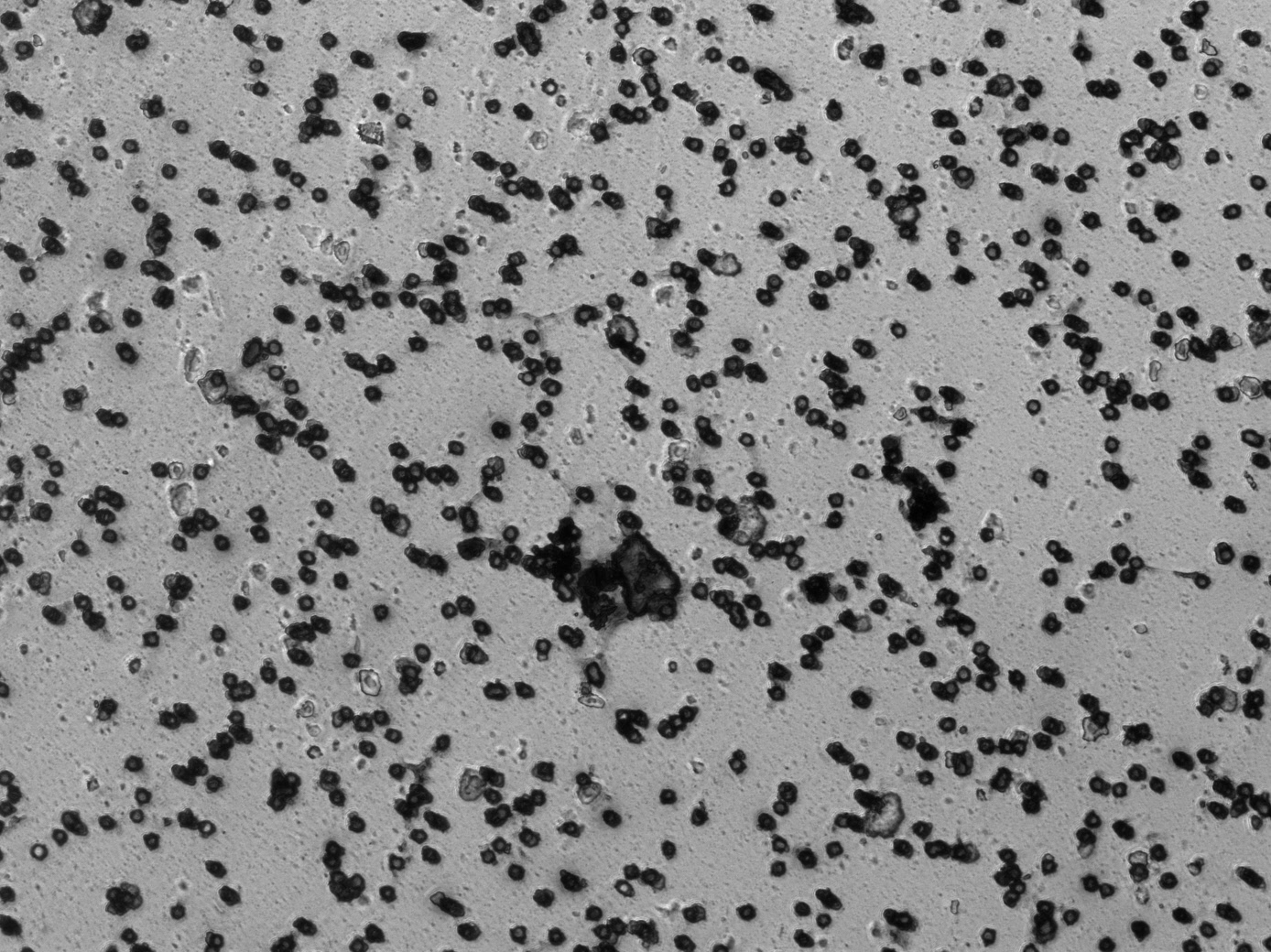

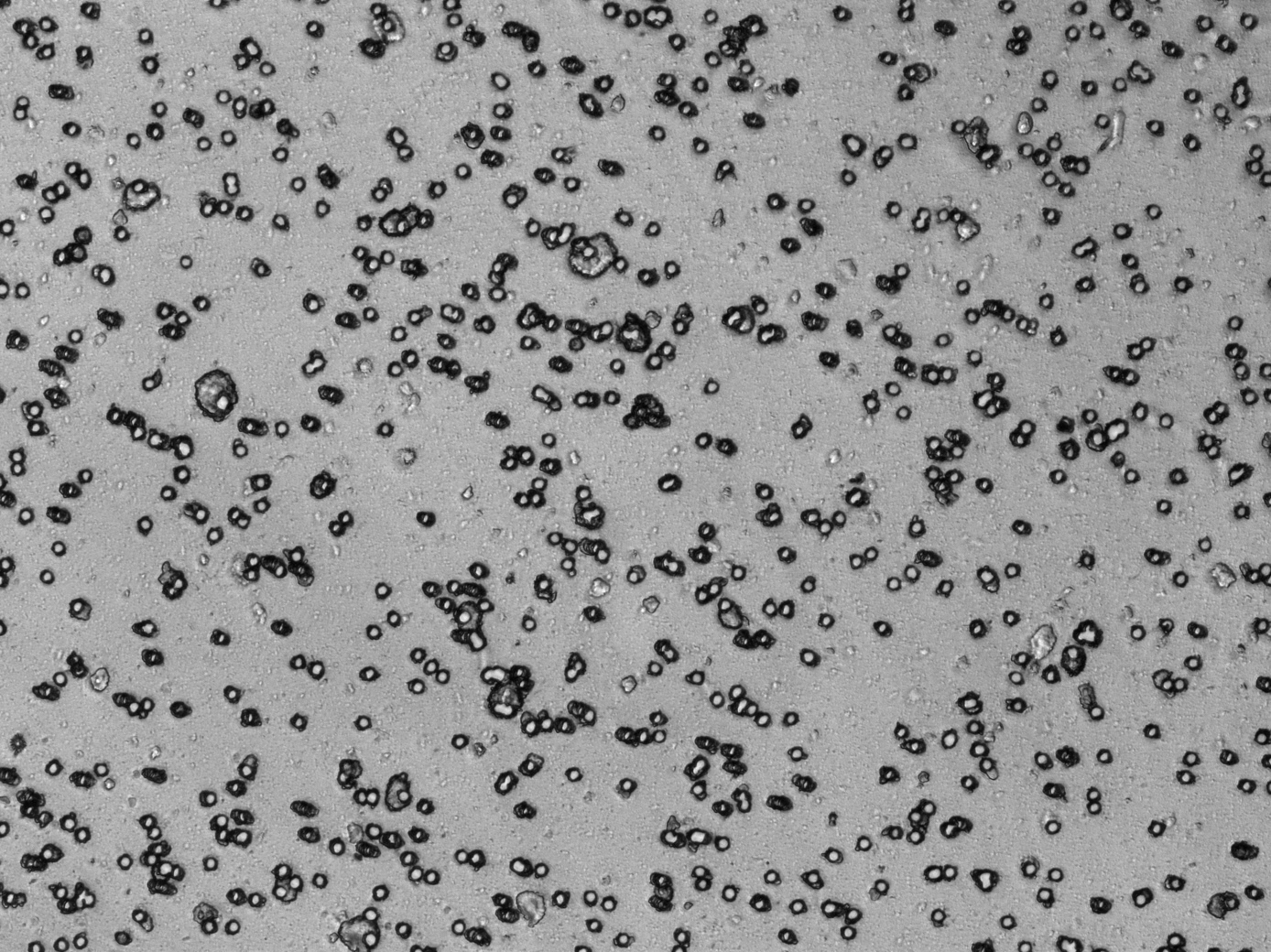

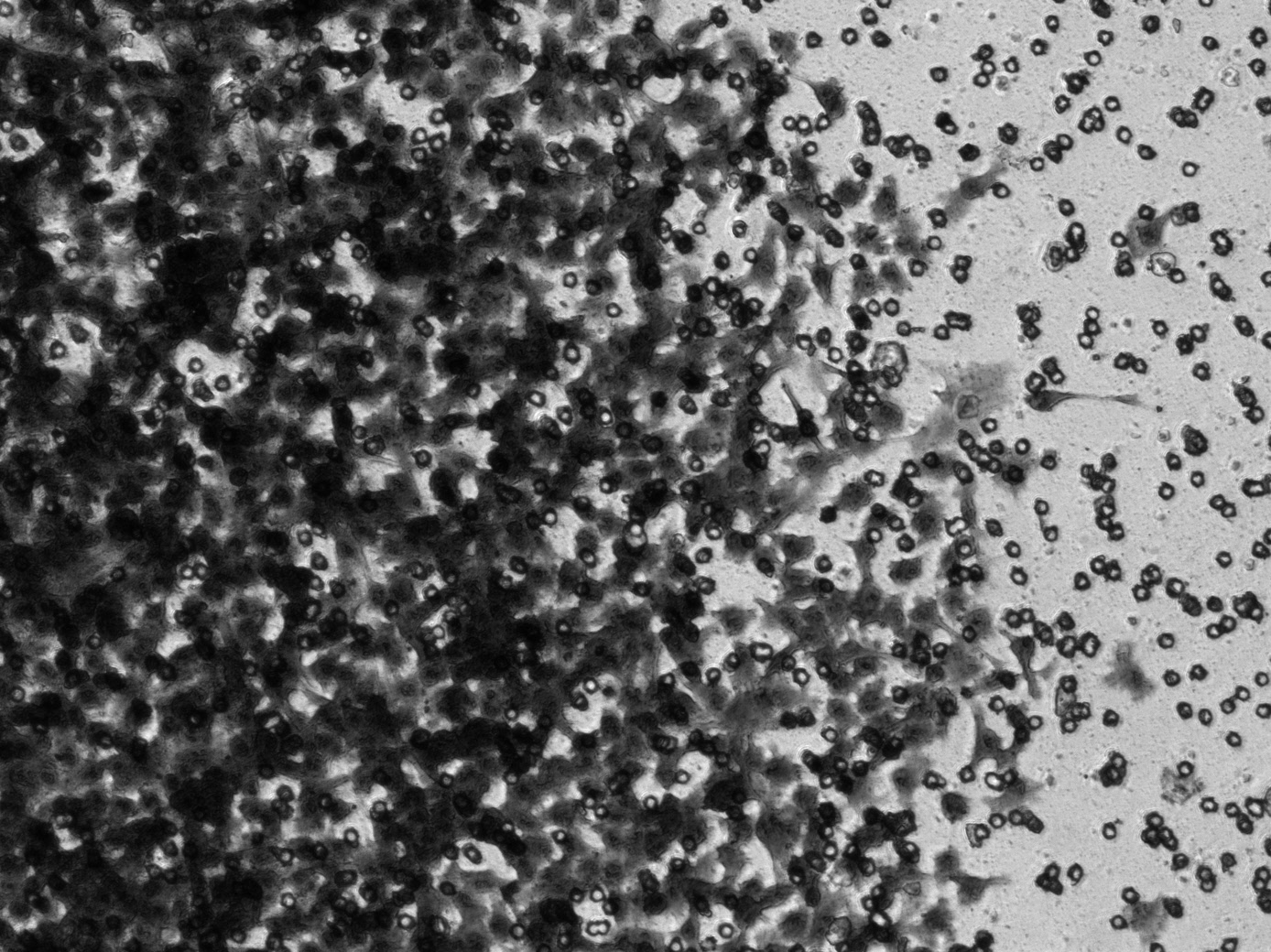


TOV21G^LI^

TOV21G^HI^

control

quercetin

**Table S3.**Alphabetic list of identified differentially-expressed proteins after performing 2D-DIGE in combination with MALDI-TOF MS spectrometry between TOV21G^LI^ and TOV21G^HI^ cells.

| Spot No. | Swiss-prot No. | Gene name | Protein name | MW | pI | Score | No. Match. Peptides | Cov. (%) | Functional ontology | Subcellular location | GH(-)+25 mg GH / GH(-) | T-test | GH(-)+50 mg GH / GH(-) | T-test | GH(+) / GH(-) | T-test | Matched peptides |
| --- | --- | --- | --- | --- | --- | --- | --- | --- | --- | --- | --- | --- | --- | --- | --- | --- | --- |
| 313 | O43175 | SERA_HUMAN | D-3-phosphoglycerate dehydrogenase | 57356 | 6.29 | 123/56 | 9/12 | 13% | Amino acid biosynthesis | Cytoplasm | -1.12 | 0.47 | -1.08 | 0.28 | -1.68 | 0.0038 | AFANLR DGKWER VVNCAR |
| 550 | P04181 | OAT_HUMAN | Ornithine aminotransferase, mitochondrial | 48846 | 6.57 | 101/56 | 8/16 | 15% | Amino acid biosynthesis | Mitochondrion | -1.33 | 0.16 | -1.32 | 0.076 | -1.87 | 0.028 | LFNYHK KWGYTVK DWDAWK |
| 396 | P25705 | ATPA_HUMAN | ATP synthase subunit alpha, mitochondrial | 59828 | 9.16 | 114/56 | 14/28 | 16% | ATP biosynthesis | Mitochondrion | -1.58 | 0.0014 | -1.56 | 0.0022 | -1.16 | 0.18 | VHGLR APGIIPR ELIIGDR |
| 784 | O00764 | PDXK_HUMAN | Pyridoxal kinase | 35308 | 5.75 | 85/56 | 6/11 | 19% | Biosynthesis | Cytoplasm | 1.05 | 0.65 | 1.15 | 0.21 | 2.11 | 0.0063 | VLSIQSHVIR GYVGNR TIQCAK |
| 253 | O60701 | UGDH_HUMAN | UDP-glucose 6-dehydrogenase | 55674 | 6.73 | 155/56 | 12/17 | 22% | Carbohydrate biosynthesis | Cytoplasm | 1.05 | 0.47 | 1.13 | 0.28 | 1.53 | 0.041 | VTVVDVNESR EVVESCR AAESIRR |
| 901 | Q14376 | GALE_HUMAN | UDP-glucose 4-epimerase | 38656 | 6.26 | 106/56 | 8/11 | 18% | Carbohydrate biosynthesis | Cytoplasm | -1.09 | N.A. | -1.16 | 0.5 | 4.1 | 0.029 | VQELTGR TWNAVLLR LKEQCGCR |
| 164 | Q66GS9 | CP135_HUMAN | Centrosomal protein of 135 kDa | 133864 | 5.87 | 61/56 | 13/27 | 10% | Centriole biogenesis | Cytoplasm | -3.87 | 9.70E-06 | -3.55 | 2.30E-05 | -8.46 | 0.00015 | CARETADLK IQQLQEK DYYKK |
| 280 | P08670 | VIME_HUMAN | Vimentin | 53676 | 5.06 | 189/56 | 18/29 | 29% | Cytoskeleton | Cytoplasm | -1.18 | 0.13 | -1.24 | 0.059 | 1.84 | 0.0017 | SVSSSSYR SYVTTSTR FANYIDK |
| 281 | P08670 | VIME_HUMAN | Vimentin | 53676 | 5.06 | 190/56 | 18/29 | 33% | Cytoskeleton | Cytoplasm | -1.37 | 0.013 | -1.37 | 0.024 | 1.75 | 0.0033 | VELQELNDR FANYIDK FLEQQNK |
| 337 | P08670 | VIME_HUMAN | Vimentin | 53676 | 5.06 | 154/56 | 16/30 | 27% | Cytoskeleton | Cytoplasm | -1.15 | N.A. | -1.08 | 0.21 | 1.63 | 0.0097 | SVSSSSYR SYVTTSTR FANYIDK |
| 353 | P08670 | VIME_HUMAN | Vimentin | 53676 | 5.06 | 134/56 | 15/27 | 32% | Cytoskeleton | Cytoplasm | -1.13 | 0.11 | -1.21 | 0.0048 | 1.57 | 0.00055 | SVSSSSYR SYVTTSTR QDVDNASLAR |
| 354 | P08670 | VIME_HUMAN | Vimentin | 53676 | 5.06 | 86/56 | 15/39 | 30% | Cytoskeleton | Cytoplasm | -1.1 | 0.15 | -1.2 | 0.0034 | 1.71 | 5.80E-05 | SYVTTSTR VELQELNDR FLEQQNK |
| 368 | P08670 | VIME_HUMAN | Vimentin | 53676 | 5.06 | 208/56 | 33/62 | 60% | Cytoskeleton | Cytoplasm | -1.23 | 0.0072 | -1.33 | 0.0019 | 2.19 | 1.10E-05 | SVSSSSYR VELQELNDR QQYESVAAK |
| 372 | P08670 | VIME_HUMAN | Vimentin | 53676 | 5.06 | 143/56 | 22/39 | 43% | Cytoskeleton | Cytoplasm | -1.2 | 0.0037 | -1.23 | 0.00084 | 1.82 | 3.50E-05 | VELQELNDR FANYIDK DNLAEDIMR |
| 430 | P05787 | K2C8_HUMAN | Keratin, type II cytoskeletal 8 | 53671 | 5.52 | 62/56 | 7/17 | 11% | Cytoskeleton | Cytoplasm | 1.61 | 0.0021 | 1.55 | 0.0073 | 2.22 | 0.00028 | VGSSNFR FASFIDK HGDDLRR |
| 435 | P05787 | K2C8_HUMAN | Keratin, type II cytoskeletal 8 | 53671 | 5.52 | 115/56 | 13/26 | 25% | Cytoskeleton | Cytoplasm | -1.12 | 0.028 | -1.25 | 0.0015 | -1.7 | 6.90E-05 | SYTSGPGSR ISSSSFSR FASFIDK |
| 490 | P05787 | K1C18_HUMAN | Keratin, type I cytoskeletal 18 | 48029 | 5.34 | 84/56 | 9/21 | 14% | Cytoskeleton | Cytoplasm | -1.24 | 0.036 | -1.14 | 0.25 | -1.91 | 0.00039 | STFSTNYR LASYLDRVR LAADDFR |
| 506 | P08670 | VIME_HUMAN | Vimentin | 53676 | 5.06 | 107/56 | 14/32 | 20% | Cytoskeleton | Cytoplasm | -2.25 | 0.039 | -2.51 | N.A. | 1.83 | 0.014 | SSVPGVR VELQELNDR FLEQQNK |
| 519 | P05783 | K1C18_HUMAN | Keratin, type I cytoskeletal 18 | 48029 | 5.34 | 109/56 | 12/20 | 23% | Cytoskeleton | Cytoplasm | -1.06 | 0.04 | -1.09 | 0.021 | -1.95 | 9.50E-05 | STFSTNYR LAADDFR KVIDDTNITR |
| 539 | P08670 | VIME_HUMAN | Vimentin | 53676 | 5.06 | 115/56 | 11/20 | 16% | Cytoskeleton | Cytoplasm | -2.15 | 0.00034 | -1.74 | 0.00036 | 1.05 | 0.55 | FANYIDKVR FANYIDK SSVPGVR |
| 568 | P60709 | ACTB_HUMAN | Actin, cytoplasmic 1 | 42052 | 5.29 | 91/56 | 8/18 | 18% | Cytoskeleton | Cytoplasm | -1.72 | 0.0052 | -1.49 | N.A. | -1.17 | 0.26 | LDLAGR IIAPPER IIAPPERK |
| 1500 | O15511 | ARPC5_HUMAN | Actin-related protein 2/3 complex subunit 5 | 16367 | 5.47 | 66/56 | 4/6 | 21% | Cytoskeleton | Cytoplasm | -1.05 | 0.66 | -1.02 | 0.83 | 1.5 | 0.0062 | NTVSSAR NPPINTK VLISFK |
| 29 | P15311 | EZRI_HUMAN | Ezrin | 69484 | 5.94 | 111/56 | 13/41 | 18% | Cytoskeleton regulation | Cytoplasm | -1.11 | 0.052 | -1.13 | 0.0055 | 1.67 | 4.10E-05 | QLFDQVVK DQWEDR IGFPWSEIR |
| 882 | O00151 | LASP1_HUMAN | LIM and SH3 domain protein 1 | 30097 | 6.61 | 93/56 | 12/26 | 35% | Cytoskeleton regulation | Cytoplasm | -1.07 | 0.21 | -1.12 | 0.022 | 1.78 | 0.00015 | ACFHCETCK MTLNMKNYK QQSELQSQVR |
| 937 | O00151 | PDLI1_HUMAN | PDZ and LIM domain protein 1 | 36505 | 6.56 | 65/56 | 5/14 | 16% | Cytoskeleton regulation | Cytoplasm | -2.23 | 0.0088 | -2.14 | 0.026 | -3.04 | 0.01 | DFEQPLAISR GDPNKPSGFR APVTK |
| 947 | P67936 | TPM4_HUMAN | Tropomyosin alpha-4 chain | 28619 | 4.67 | 107/56 | 10/30 | 22% | Cytoskeleton regulation | Cytoplasm | 1.26 | 0.24 | 1.05 | 0.68 | 1.57 | 0.0065 | AEGDVAALNR AEGDVAALNRR HIAEEADR |
| 1482 | P23528 | COF1_HUMAN | Cofilin-1 | 18719 | 8.22 | 90/56 | 8/20 | 39% | Cytoskeleton regulation | Cytoplasm | -1.12 | 0.38 | 1.02 | 0.77 | 1.75 | 0.0046 | VFNDMK MIYASSK MLPDKDCR |
| 1509 | P23528 | COF1_HUMAN | Cofilin-1 | 18719 | 8.22 | 105/56 | 12/32 | 48% | Cytoskeleton regulation | Cytoplasm | -1.02 | 0.57 | 1.06 | 0.087 | 1.99 | 4.4E-05 | VFNDMK SSTPEEVK NIILEEGK |
| 1515 | P60981 | DEST_HUMAN | Destrin | 18950 | 8.06 | 99/56 | 8/15 | 36% | Cytoskeleton regulation | Cytoplasm | -1.15 | 0.026 | -1.12 | 0.04 | 1.54 | 0.00041 | IFYDMK IFYDMKVR HFVGMLPEK |
| 1525 | Q9Y2B0 | CNPY2_HUMAN | Protein canopy homolog 2 | 20981 | 4.81 | 73/56 | 6/14 | 28% | Cytoskeleton regulation | ER | -1.54 | 0.013 | -1.22 | 0.13 | -1.37 | 0.061 | TIQMGSFR SQDLHCGACR KNYVR |
| 694 | Q9Y6N5 | SQRD_HUMAN | Sulfide:quinone oxidoreductase, mitochondrial | 50214 | 9.18 | 58/56 | 7/18 | 11% | Electron transport | Mitochondrion | -2.9 | 0.0076 | -2.36 | 0.0055 | -4.37 | 0.00082 | KNLIEVR TISVIMK LSMYLMK |
| 700 | Q9Y6N5 | SQRD_HUMAN | Sulfide:quinone oxidoreductase, mitochondrial | 50214 | 9.18 | 73/56 | 6/14 | 11% | Electron transport | Mitochondrion | -2.48 | 8.30E-06 | -2.29 | 9.10E-06 | -32.57 | 0.00016 | AQLFACLLR TWKALQDFK KNLIEVR |
| 1090 | Q16775 | GLO2_HUMAN | Hydroxyacylglutathione hydrolase, mitochondrial | 34240 | 8.35 | 58/56 | 5/16 | 12% | Glutathione biosynthesis | Mitochondrion | -1.27 | 0.0014 | -1.37 | 0.055 | -1.61 | 0.0018 | VYGGDDR LPPDTR LAWAKEK |
| 300 | P11413 | G6PD_HUMAN | Glucose-6-phosphate 1-dehydrogenase | 59675 | 6.39 | 174/56 | 24/39 | 39% | Glycolysis | Cytoplasm | 1.07 | 0.032 | 1.09 | 0.02 | -2.04 | 0.0011 | TQVCGILR QSEPFFK LEDFFAR |
| 462 | P06733 | ENOA_HUMAN | Alpha-enolase | 47481 | 7.01 | 64/56 | 7/20 | 11% | Glycolysis | Cytoplasm | -1.03 | 0.43 | -1.02 | 0.8 | -1.86 | 0.00053 | EIFDSR GVPLYR TGAPCR |
| 686 | P04075 | ALDOA_HUMAN | Fructose-bisphosphate aldolase A | 39851 | 8.3 | 67/56 | 7/22 | 14% | Glycolysis | Cytoplasm | -1.69 | 4.10E-05 | -1.54 | 0.00064 | -3.1 | 0.0068 | RFYR QLLLTADDR VLAAVYK |
| 877 | P07195 | LDHB_HUMAN | L-lactate dehydrogenase B chain | 36900 | 5.71 | 91/56 | 7/9 | 16% | Glycolysis | Cytoplasm | -1.03 | 0.35 | -1.03 | 0.26 | 1.68 | 0.00059 | QQEGESR FIIPQIVK SADTLWDIQK |
| 1098 | P18669 | PGAM1_HUMAN | Phosphoglycerate mutase 1 | 28900 | 6.67 | 103/56 | 8/15 | 22% | Glycolysis | Cytoplasm | 2.27 | 8.30E-06 | 2.2 | 1.30E-06 | 1.9 | 0.00043 | HGESAWNLENR VLIAAHGNSLR HYGGLTGLNK |
| 1666 | P09382 | LEG1_HUMAN | Galectin-1 | 15048 | 5.34 | 84/56 | 5/13 | 39% | Growth regulation | Secreted | 1.06 | 0.003 | 1.04 | 0.23 | 2.11 | 2.7E-06 | SFVLNLGK VRGEVAPDAK LPDGYEFK |
| 67 | Q8IX19 | MCEM1_HUMAN | Mast cell-expressed membrane protein 1 | 21443 | 9.03 | 70/56 | 5/15 | 14% | immunoresponse | Plasma Membrane | -1.45 | 0.00028 | -1.52 | 0.0002 | -1.19 | 0.017 | ELLGFK ILEVLQK EVEEIYK |
| 521 | P42765 | THIM_HUMAN | 3-ketoacyl-CoA thiolase, mitochondrial | 42354 | 8.32 | 80/56 | 6/12 | 14% | Lipid catabolism | Mitochondrion | -1.59 | 0.0092 | -1.34 | 0.052 | -1.76 | 0.17 | TPFGAYGGLLK YALQSQQR HNFTPLAR |
| 628 | Q9BWD1 | THIC_HUMAN | Acetyl-CoA acetyltransferase, cytosolic | 41838 | 6.47 | 72/56 | 6/16 | 13% | Lipid catabolism | Cytoplasm | 1.44 | 0.00036 | 1.62 | 8.7E-05 | -1.03 | 0.67 | APHLAYLR KWQVSR VAVLSQNR |
| 640 | P24752 | THIL_HUMAN | Acetyl-CoA acetyltransferase, mitochondrial | 45456 | 8.98 | 78/56 | 7/18 | 13% | Lipid catabolism | Mitochondrion | -1.27 | 0.069 | -1.27 | 0.064 | -1.81 | 0.048 | GSTPYGGVK DGLTDVYNK LNVTPLAR |
| 724 | O00154 | BACH_HUMAN | Cytosolic acyl coenzyme A thioester hydrolase | 42454 | 8.85 | 104/56 | 10/20 | 18% | Lipid catabolism | Cytoplasm | -1.19 | 0.16 | -1.29 | 0.077 | -1.7 | 0.0094 | HCNSQNGER MTFTSNK FEEGKGR |
| 1280 | Q15102 | PA1B3_HUMAN | Platelet-activating factor acetylhydrolase IB subunit gamma | 25832 | 6.33 | 74/56 | 6/17 | 21% | Lipid catabolism | Cytoplasm | -1.81 | 0.0021 | -1.66 | 0.078 | -4.25 | 0.024 | WMSLHHR VVVLGLLPR ALHSLLLR |
| 1282 | P62826 | RAN_HUMAN | GTP-binding nuclear protein Ran | 24579 | 7.01 | 86/56 | 8/33 | 35% | Nucleocytoplasmic transport | Nucleus | -1.39 | 0.00012 | -1.4 | 7.80E-05 | -2.28 | 0.00018 | HLTGEFEK NVPNWHR SIVFHR |
| 206 | P31939 | PUR9_HUMAN | Bifunctional purine biosynthesis protein PURH | 65089 | 7.12 | 74/56 | 8/20 | 10% | Nucleotide biosynthesis | Cytoplasm | 1.25 | 0.014 | 1.13 | 0.22 | 1.86 | 0.05 | TGLVEFAR LDFNLIR DLPESALR |
| 294 | O60701 | CAP1_HUMAN | Adenylyl cyclase-associated protein 1 | 52325 | 8.24 | 106/56 | 11/20 | 15% | Nucleotide biosynthesis | Cytoplasm | -1.17 | 0.036 | -1.16 | 0.083 | 1.59 | 0.00064 | EVITFR HVDWVK AQSGPVR |
| 526 | Q96MA6 | KAD8_HUMAN | Adenylate kinase 8 | 55233 | 5.77 | 70/56 | 5/17 | 9% | Nucleotide biosynthesis | Cytoplasm | -1.08 | 0.19 | -1.21 | 0.081 | 1.7 | 0.024 | DLDQAHLLNR LGYNPNR IDPVTGER |
| 1422 | P33316 | DUT_HUMAN | Deoxyuridine 5'-triphosphate nucleotidohydrolase, mitochondrial | 26832 | 9.46 | 65/56 | 4/13 | 15% | Nucleotide biosynthesis | Mitochondrion | 1.82 | 0.017 | 2.11 | 0.0093 | -1.17 | 0.49 | LSEHATAPTR IAQLICER GSGGFGSTGK |
| 341 | P34897 | GLYM_HUMAN | Serine hydroxymethyltransferase, mitochondrial | 56414 | 8.76 | 161/56 | 16/19 | 21% | One carbon metabolism | Mitochondrion | 1.05 | 0.59 | 1.11 | 0.18 | -1.57 | 0.02 | YSEGYPGKR SGLIFYR EIPYTFEDR |
| 1692 | P04080 | CYTB_HUMAN | Cystatin-B | 11190 | 6.69 | 89/56 | 5/12 | 39% | Protease inhibitor | Cytoplasm | -1.02 | 0.69 | -1.15 | 0.05 | 1.79 | 0.003 | SQVVAGTNYFIK VHVGDEDFVHLR AKHDELTYF |
| 509 | O00231 | PSD11_HUMAN | 26S proteasome non-ATPase regulatory subunit 11 | 47719 | 6.08 | 94/56 | 9/21 | 18% | Protein degradation | Cytoplasm | -1.47 | 0.0032 | -1.4 | 0.0013 | -3.15 | 0.0058 | AQSLLSTDR YVRPFLNSISK YMLLCK |
| 959 | P25786 | PSA1_HUMAN | Proteasome subunit alpha type-1 | 29822 | 6.15 | 82/56 | 9/20 | 34% | Protein degradation | Cytoplasm | -2.6 | 4.50E-05 | -2.51 | 7.10E-05 | -12.32 | 0.0082 | QGSATVGLK AQSELAAHQK TQIPTQR |
| 532 | O75592 | MYCB2_HUMAN | E3 ubiquitin-protein ligase MYCBP2 | 517856 | 6.63 | 58/56 | 18/25 | 4% | Protein degradation | Cytoplasm | -1.83 | 0.00047 | -1.65 | 0.00013 | -15.85 | 0.002 | LNDETIK FHPELSK THQRQVFK |
| 1154 | P04632 | CPNS1_HUMAN | Calpain small subunit 1 | 28469 | 5.05 | 100/56 | 9/19 | 21% | Protein degradation | Cytoplasm | -1.11 | 0.17 | -1.08 | 0.3 | 1.5 | 0.036 | QFRR VVTRHPDLK WQAIYK |
| 41 | O15212 | PFD6_HUMAN | Prefoldin subunit 6 | 14574 | 8.83 | 64/56 | 6/20 | 37% | Protein folding | Cytoplasm | -1.57 | 0.00031 | -1.61 | 0.00022 | -4.91 | 0.00049 | MAELIQK MAELIQK RYESQLR |
| 45 | P11021 | GRP78_HUMAN | 78 kDa glucose-regulated protein | 72402 | 5.07 | 92/56 | 10/15 | 14% | Protein folding | ER | -1.9 | 8.8E-05 | -1.99 | 0.00019 | -1.5 | 0.021 | VEIIANDQGNR ALSSQHQAR |
| 73 | P11021 | GRP78_HUMAN | 78 kDa glucose-regulated protein | 72402 | 5.07 | 85/56 | 9/18 | 14% | Protein folding | ER | -2.34 | 9.90E-05 | -2.32 | 3.50E-06 | -2.04 | 1.40E-05 | VEIIANDQGNR FLPFK ALSSQHQAR |
| 90 | P11021 | GRP78_HUMAN | 78 kDa glucose-regulated protein | 72402 | 5.07 | 149/56 | 16/23 | 21% | Protein folding | ER | -2.68 | 1.30E-07 | -2.51 | 3.40E-07 | -2.77 | 4.70E-07 | VEIIANDQGNR ALSSQHQAR VYEGERPLTK |
| 146 | P08107 | HSP71_HUMAN | Heat shock 70 kDa protein 1A/1B | 70294 | 5.48 | 80/56 | 8/17 | 15% | Protein folding | Cytoplasm | 1.07 | 0.17 | 1.12 | 0.018 | -1.53 | 0.0026 | VEIIANDQGNR DAGVIAGLNVLR LLQDFFNGR |
| 297 | Q02790 | FKBP4_HUMAN | Peptidyl-prolyl cis-trans isomerase FKBP4 | 52057 | 5.35 | 113/56 | 12/17 | 16% | Protein folding | Cytoplasm | 1.33 | 0.16 | -1.16 | 0.46 | 3.31 | 0.0013 | DKFSFDLGK FSFDLGK QALLQYK |
| 892 | Q13765 | NACA_HUMAN | Nascent polypeptide-associated complex subunit alpha | 23370 | 4.52 | 57/56 | 5/10 | 22% | Protein folding | Cytoplasm | 2.05 | 0.0068 | 1.83 | 0.0027 | -1.13 | 0.38 | QVTGVTR QVTGVTR DIELVMSQANVSR |
| 1187 | P04792 | HSPB1_HUMAN | Heat shock protein beta-1 | 22826 | 5.98 | 135/56 | 10/21 | 38% | Protein folding | Cytoplasm | -1.38 | 0.0031 | -1.32 | 0.003 | 1.71 | 0.00028 | VPFSLLR DWYPHSR QLSSGVSEIR |
| 756 | P53004 | BIEA_HUMAN | Biliverdin reductase A | 33692 | 6.06 | 68/56 | 6/15 | 19% | Redox regulation | Cytoplasm | -1.95 | 0.034 | -1.82 | 0.053 | -4.19 | 0.0012 | FGVVVVGVGR YLSFHFK LLGQFSEK |
| 1139 | P30041 | PRDX6_HUMAN | Peroxiredoxin-6 | 25513 | 6 | 170/56 | 13/26 | 46% | Redox regulation | Cytoplasm | -1.12 | 0.012 | -1.11 | 0.0089 | -1.59 | 7.00E-05 | LAPEFAK VATPVDWK GVFTK |
| 1209 | P30048 | PRDX3_HUMAN | Thioredoxin-dependent peroxide reductase, mitochondrial | 28017 | 7.67 | 73/56 | 7/17 | 26% | Redox regulation | Mitochondrion | -1.39 | 0.035 | -1.51 | 0.0038 | -1.13 | 0.096 | GTAVVNGEFK DLSLDDFK SVEETLR |
| 1223 | P30048 | PRDX3_HUMAN | Thioredoxin-dependent peroxide reductase, mitochondria | 28017 | 7.67 | 70/56 | 5/12 | 19% | Redox regulation | Mitochondrion | 1.21 | 0.82 | -1.03 | 0.68 | 1.51 | 0.0086 | DLSLDDFK HLSVNDLPVGR SVEETLR |
| 1226 | P09211 | GSTP1_HUMAN | Glutathione S-transferase P | 23569 | 5.43 | 83/56 | 6/9 | 31% | Redox regulation | Cytoplasm | -1.1 | 0.026 | -1.1 | 0.0057 | 1.93 | 0.00013 | ASCLYGQLPK PPYTVVYFPVR MLLADQGQSWK |
| 1228 | P09212 | GSTP2_HUMAN | Glutathione S-transferase P | 23569 | 5.43 | 76/56 | 5/9 | 18% | Redox regulation | Cytoplasm | -1.03 | 0.79 | -1.12 | 0.28 | 1.92 | 0.013 | PPYTVVYFPVR ASCLYGQLPK TLGLYGK |
| 1268 | Q06830 | PRDX1_HUMAN | Peroxiredoxin-1 | 22324 | 8.27 | 199/56 | 13/18 | 53% | Redox regulation | Cytoplasm | -1.14 | 0.37 | 1.05 | 0.7 | 4.41 | 0.0016 | IGHPAPNFK ADEGISFR GLFIIDDK |
| 961 | A8K979 | ERI2_HUMAN | ERI1 exoribonuclease 2 | 78549 | 9.12 | 68/56 | 9/24 | 8% | RNA degradation | Cytoplasm | -1.6 | N.A. | -1.33 | 0.22 | 3.37 | 0.0068 | KNFSILAR NIINPHEK CCGYFK |
| 248 | P31150 | GDIA_HUMAN | Rab GDP dissociation inhibitor alpha | 51177 | 5 | 80/56 | 8/10 | 12% | Signal transduction | Cytoplasm | -1.58 | 0.0033 | -1.38 | 0.0096 | -1.16 | 0.19 | MLLYTEVTR VVEGSFVYK LYSESLAR |
| 528 | Q9UQ80 | PA2G4_HUMAN | Proliferation-associated protein 2G4 | 44101 | 6.13 | 106/56 | 18/31 | 31% | Signal transduction | Cytoplasm | -1.58 | 0.0035 | -1.57 | 0.016 | -3.41 | 0.09 | MGGDIANR SDQDYILK AFFSEVER |
| 541 | O15021 | MAST4_HUMAN | Microtubule-associated serine/threonine-protein kinase 4 | 286426 | 8.85 | 59/56 | 10/14 | 3% | Signal transduction | Cytoplasm | -1.29 | 0.00042 | -1.22 | 0.0015 | -4.14 | 1.30E-05 | SFSCLNR ELSLPRR RVQSEEK |
| 866 | P53004 | ANXA2_HUMAN | Annexin A2 | 38808 | 7.57 | 67/56 | 9/22 | 17% | Signal transduction | Secreted | 1.09 | 0.42 | 1.1 | 0.2 | 1.77 | 0.0066 | AYTNFDAER QDIAFAYQR TPAQYDASELK |
| 868 | P07355 | ANXA2_HUMAN | Annexin A2 | 38808 | 7.57 | 159/56 | 20/37 | 44% | Signal transduction | Secreted | -1.01 | 0.75 | -1.04 | 0.14 | 1.62 | 0.00026 | AYTNFDAER QDIAFAYQR TPAQYDASELK |
| 902 | P04083 | ANXA1_HUMAN | Annexin A1 | 38918 | 6.57 | 90/56 | 13/34 | 42% | Signal transduction | Plasma membrane | -1.17 | 0.008 | -1.2 | 0.0023 | -1.59 | 0.00059 | TPAQFDADELR VYREELK NALLSLAK |
| 912 | P04083 | ANXA1_HUMAN | Annexin A1 | 38918 | 6.57 | 91/56 | 11/33 | 32% | Signal transduction | Plasma membrane | -1.15 | 0.24 | -1.19 | 0.036 | -1.54 | 0.00041 | ALYEAGER SYPQLR VLDLELK |
| 956 | P09525 | ANXA4_HUMAN | Annexin A4 | 36088 | 5.84 | 124/56 | 14/19 | 37% | Signal transduction | Cytoplasm | -1.06 | 0.17 | 1.01 | 0.98 | 1.9 | 0.0018 | TPEEIRR SLEDDIR SDTSFMFQR |
| 989 | P63244 | GBLP_HUMAN | Guanine nucleotide-binding protein subunit beta-2-like 1 | 35511 | 7.6 | 90/56 | 7/17 | 20% | Signal transduction | Plasma membrane | 1.01 | 0.99 | 1.05 | 0.61 | -1.76 | 0.0065 | LWNTLGVCK VWNLANCK IWDLEGK |
| 1862 | P63244 | GBLP_HUMAN | Guanine nucleotide-binding protein subunit beta-2-like 1 | 35511 | 7.6 | 229/56 | 20/36 | 52% | Signal transduction | Plasma membrane | 1.02 | 0.61 | 1 | 0.84 | -1.6 | 0.0019 | TEQMTLR DKTIIMWK TIIMWK |
| 818 | Q9UK10 | ZN225_HUMAN | Zinc finger protein 225 | 84927 | 9.18 | 58/56 | 11/33 | 14% | Transcriptional regulation | Nucleus | -1.22 | 0.004 | -1.05 | 0.64 | -5.11 | 0.0096 | EVMLENFR FWMMETATQR HSMVHMR |
| 1417 | P62633 | CNBP_HUMAN | Cellular nucleic acid-binding protein | 20704 | 8 | 82/56 | 6/17 | 31% | Transcriptional regulation | Cytoplasm | 1.41 | 0.38 | 1.37 | 0.25 | -4.22 | 0.0013 | SSNECFK SGHWAR TSEVNCYR |
| 174 | P49590 | SYHM_HUMAN | Probable histidine--tRNA ligase, mitochondrial | 57593 | 8.52 | 77/56 | 14/34 | 19% | Translational regulation | Mitochodrion | -2.77 | 2.10E-06 | -2.51 | 4.50E-08 | -27.25 | 3.00E-05 | HEMVVKK RESPTIVQGR AEMLYKNNPK |
| 500 | O00303 | EIF3F_HUMAN | Eukaryotic translation initiation factor 3 subunit F | 37654 | 5.24 | 85/56 | 9/19 | 21% | Translational regulation | Cytoplasm | -1.55 | 0.0015 | -1.47 | 0.0022 | -1.14 | 0.12 | MAAPVLLR VSVPRWER LVYLNEAWKR |
| 1246 | P13693 | TCTP_HUMAN | Translationally-controlled tumor protein | 19697 | 4.84 | 74/56 | 9/23 | 36% | Translational regulation | Cytoplasm | -1.27 | 0.11 | -1.15 | 0.21 | 1.53 | 0.039 | YIKDYMK LEEQRPER HILANFK |
| 1346 | P13693 | TCTP_HUMAN | Translationally-controlled tumor protein | 19697 | 4.84 | 83/56 | 8/21 | 32% | Translational regulation | Cytoplasm | 1.53 | 1.50E-05 | 1.47 | 0.0048 | -1.07 | 0.14 | LEEQRPER HILANFK EDGVTPYMIFFK |
| 356 | Q13228 | SBP1_HUMAN | Selenium-binding protein 1 | 52928 | 5.93 | 66/56 | 8/17 | 16% | Vesicle transport | Golgi apparatus | -1.74 | 5.70E-05 | -1.81 | 0.00036 | -1.09 | 0.41 | SPQYCQVIHR LVLPSLISSR DGLIPLEIR |
